# Wheat TILLING Mutants Show That the Vernalization Gene *VRN1* Down-Regulates the Flowering Repressor *VRN2* in Leaves but Is Not Essential for Flowering

**DOI:** 10.1371/journal.pgen.1003134

**Published:** 2012-12-13

**Authors:** Andrew Chen, Jorge Dubcovsky

**Affiliations:** 1 Department of Plant Sciences, University of California Davis, Davis, California, United States of America; 2 Howard Hughes Medical Institute, Chevy Chase, Maryland, United States of America; 3 Gordon and Betty Moore Foundation, Palo Alto, California, United States of America; Commonwealth Scientific and Industrial Research Organisation, Australia

## Abstract

Most of the natural variation in wheat vernalization response is determined by allelic differences in the MADS-box transcription factor *VERNALIZATION1* (*VRN1*). Extended exposures to low temperatures during the winter (vernalization) induce *VRN1* expression and promote the transition of the apical meristem to the reproductive phase. In contrast to its Arabidopsis homolog (*APETALA1*), which is mainly expressed in the apical meristem, *VRN1* is also expressed at high levels in the leaves, but its function in this tissue is not well understood. Using tetraploid wheat lines with truncation mutations in the two homoeologous copies of *VRN1* (henceforth *vrn1*-null mutants), we demonstrate that a central role of *VRN1* in the leaves is to maintain low transcript levels of the *VRN2* flowering repressor after vernalization. Transcript levels of *VRN2* were gradually down-regulated during vernalization in both mutant and wild-type genotypes, but were up-regulated after vernalization only in the *vrn1*-null mutants. The up-regulation of *VRN2* delayed flowering by repressing the transcription of *FT*, a flowering-integrator gene that encodes a mobile protein that is transported from the leaves to the apical meristem to induce flowering. The role of *VRN2* in the delayed flowering of the *vrn1*-null mutant was confirmed using double *vrn1-vrn2-*null mutants, which flowered two months earlier than the *vrn1*-null mutants. Both mutants produced normal flowers and seeds demonstrating that *VRN1* is not essential for wheat flowering, which contradicts current flowering models. This result does not diminish the importance of *VRN1* in the seasonal regulation of wheat flowering. The up-regulation of *VRN1* during winter is required to maintain low transcript levels of *VRN2*, accelerate the induction of *FT* in the leaves, and regulate a timely flowering in the spring. Our results also demonstrate the existence of redundant wheat flowering genes that may provide new targets for engineering wheat varieties better adapted to changing environments.

## Author Summary

Crop yields are strongly associated with flowering time, therefore a precise understanding of the mechanisms involved in the regulation of flowering is required to engineer varieties adapted to new or changing environments. In wheat, most of the natural variation in flowering time is determined by *VERNALIZATION1* (*VRN1*), a gene responsible for the transition of the apical meristem from the vegetative to the reproductive phase. Extended exposures to low temperatures during winter (vernalization) induce *VRN1* expression, which promotes flowering in the spring. *VRN1* is expressed in the apices and in the leaves, but its role in the leaves is not well understood. Using two sets of *VRN1* knock-out mutants, we demonstrate that a central role of *VRN1* in the leaves is to maintain low transcript levels of the *VRN2* flowering repressor, which allows the production of the mobile FT protein (florigen) required to initiate flowering. Both sets of *VRN1* knock-out mutants flowered very late but, eventually, produced normal flowers and seeds, which demonstrates that *VRN1* is not essential for wheat flowering. This last result also demonstrates the existence of redundant flowering genes that could provide new targets for engineering flowering time in wheat.

## Introduction

The temperate grasses, which include economically important species such as wheat, barley, rye, and oats, are well-adapted to cold winters. Most of these species require a prolonged period of cold treatment for timely flowering, a process referred to as vernalization. This requirement delays the initiation of the reproductive phase and protects the sensitive floral meristems from frost damage during the winter. It also contributes to the precise adjustment of flowering time to seasonal changes, which is important to maximize seed production. Therefore, a better understanding of the mechanisms involved in the regulation of wheat flowering can contribute to the engineering of high yielding varieties adapted to changing environments.

The cloning of the three main wheat vernalization genes, *VRN1* [1]–[3], *VRN2* [4] and *VRN3* [5], and the characterization of their natural allelic variation [6]–[11] provided an important first step in our understanding of this regulatory pathway. However, the mechanisms involved in the interactions among these genes are still controversial [12]–[14]. To facilitate the discussion of these complex interactions, the information available for these three regulatory genes is presented first.

**Figure 1:**
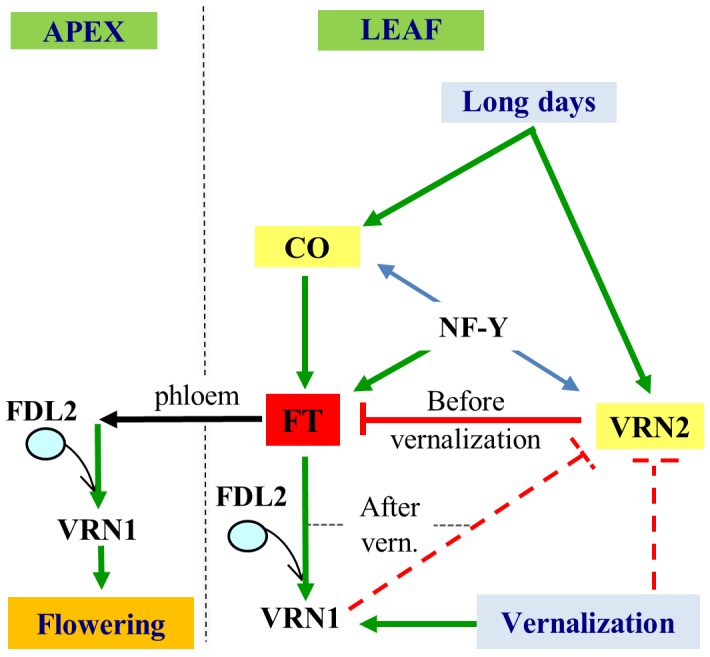
Effect of photoperiod and vernalization on wheat flowering time. During the fall, VRN2 competes successfully with CO (photoperiod pathway, *FT* promoter) for interactions with the NF-Y transcription factors, resulting in the down-regulation of *FT* transcription in the leaves [15]. This precludes flowering in the fall. Vernalization induces *VRN1* and down-regulates *VRN2* transcription in the leaves. The presence of VRN1 after the winter is important to maintain the down-regulation of *VRN2* during the spring. In the absence of *VRN2, FT* transcription is up-regulated and the encoded FT protein is transported through the phloem to the stem apical meristem. FT then interacts with FDL2 [18] to up-regulate *VRN1* transcripts to the levels required for the transition to the reproductive phase. Dashed red lines indicate interactions demonstrated in this study.

The *VRN3* gene is the main integrator of the vernalization and photoperiod signals in the temperate grasses [15] (Figure 1). This gene encodes a RAF kinase inhibitor–like protein with high similarity to Arabidopsis protein FLOWERING LOCUS T (FT) [5] and will therefore, be designated as FT hereafter. In Arabidopsis, *FT* transcription is induced by long days in the leaves and the encoded protein travels through the phloem to the stem apical meristem [16]. There, FT interacts with the bZIP transcription factor FD and up-regulates the expression of the meristem identity gene *APETALA1* (*AP1*), which leads to the transition of the stem apical meristem from the vegetative to the reproductive phase [17]. A similar interaction has been observed in wheat, where the homologous FT protein interacts with an FD-like protein (FDL2) that has the ability to bind *in vitro* the promoter of *VRN1,* the wheat homolog of *AP1* [18] (Figure 1).

The insertion of a repetitive element in the *FT* promoter in the wheat variety Hope results in the overexpression of *FT* and early flowering. Transformation of a winter wheat with this *FT* allele results in accelerated flowering even in the absence of vernalization, which suggests that high *FT* transcript levels are sufficient to overcome the vernalization requirement [5]. Furthermore, transcript levels of different wheat and barley *FT* alleles correlate well with flowering time, which suggests that the amount of *FT* transcript in the leaves is critical for the regulation of flowering time in the temperate cereals [5].

*FT* and upstream genes of the photoperiod pathway are well conserved between Arabidopsis and the temperate cereals, but the vernalization genes in these species are very different. The main Arabidopsis vernalization genes *FLOWERING LOCUS C* (*FLC*) and *FRIGIDA* (*FRI*) have not been detected in the temperate cereals, and similarly, the central flowering repressor *VRN2* has not been detected in Arabidopsis [4]. Despite the fact that they belong to different classes of proteins, VRN2 and FLC both repress *FT* and prevent flowering until the plants are vernalized (Figure 1) [5], [19]. Recent studies suggest that the negative regulation of *FT* transcription by *VRN2* in the temperate cereals is mediated by the competition between VRN2 and the photoperiod protein CONSTANS (CO, a promoter of *FT* expression) for binding with a common set of NF-Y transcription factors [15] (Figure 1). NF-Y transcription factors have been shown to be involved in *FT* activation in Arabidopsis [20], [21].

The *VRN2* locus includes two tandemly duplicated CCT domain (*CONSTANS, CO-like*, and *TOC1*) genes, *ZCCT1* and *ZCCT2*, which function as long day flowering repressors [4]. Simultaneous deletions or non-functional mutations in all *ZCCT* genes result in a spring growth habit in both barley and wheat [4], [11]. In the commercial tetraploid wheat varieties studied thus far (including the variety ‘Kronos’ used in this study), *ZCCT-A1*, *ZCCT-A2*, and *ZCCT-B1* all have natural deleterious mutations in the CCT domain and the only functional copies are the two similar *ZCCT-B2* genes present in the *VRN-B2* locus [11]. Therefore, a natural deletion including both *ZCCT-B2* genes was sufficient to generate a tetraploid wheat with spring growth habit [11]. Indirect evidence suggests that *VRN2* transcription is repressed in the spring by the up-regulation of VRN1, closing a positive feedback regulatory loop that is central for the precise regulation of flowering time in the temperate cereals (Figure 1) [22].

The wheat *VRN1* gene encodes a MADS-box transcription factor closely related to the three paralogous Arabidopsis meristem identity genes *AP1, CAULIFLOWER* (*CAL*) and *FRUITFULL* (*FUL*) [1], [3]. *VRN1* transcripts are significantly up-regulated during vernalization, both under long and short days. Since *FT* and *VRN2* transcript levels are undetectable under short days [23], [24], it was concluded that *VRN1* is a direct target of vernalization. This conclusion agrees with the observation that vernalization promotes an active chromatin state by increasing levels of histone 3 lysine 4 trimethylation (H3K4me3) and decreasing H3K27me3 in *VRN1* regulatory regions but not in those of *VRN2* or *FT* [25].

In the temperate cereals there are two additional MADs-box genes similar to *VRN1* designated as *FUL2* ( = *HvMADS8 = OsMADS15*) and *FUL3* ( = *HvMADS3 = OsMADS18*) [26]. The duplications that gave rise to these three paralogous genes in wheat are independent from the duplications in Arabidopsis since they occurred after the monocot-dicot divergence [1]. Therefore, the sub-functionalization of the duplicated meristem identity genes was also independent in these two lineages. In Arabidopsis, *AP1* and *CAL* transcripts are mostly confined to the developing flowers [27] whereas *FUL* transcripts are detected both in apices and leaves but at low levels [28]. In contrast, high levels of *VRN1* have been observed in the leaves of the temperate grasses before the emergence of spikelet primordia, which suggests that *VRN1* is part of an early signal involved in the transition from the vegetative to reproductive stages [1], [29], [30].

In Arabidopsis, all three paralogs have retained meristem identity functions, and only the triple *ap1-cal-ful* mutant is unable to form flowers [31]. In rice, in addition to the *AP1/FUL2/FUL3* homologs a fourth MADS-box gene (*OsMADS34* = *PAP2*) needs to be deleted to abolish the transition of the shoot apical meristem to the reproductive stage [32]. In contrast, diploid wheat radiation mutants lacking the *VRN1* gene are unable to flower, which suggests that *VRN1* is essential for wheat flowering [33]. These mutants, designated *maintained vegetative phase* (*mvp*) showed an unexpected down-regulation of *FT* transcript levels [13], that was not predicted by current flowering models (Figure 1). However, a more recent study showed that the radiation deletions that eliminated *VRN1* were much larger than originally described and included the wheat ortholog of *PAP2* (*AGLG1*), the *PHYTOCHROME C* (*PHYC*) and several other linked genes. Therefore, the *mvp* mutant results are open to alternative interpretations that are at the center of conflicting flowering models in the temperate grasses [13], [14].

To determine the specific role of *VRN1* in the induction of flowering and in the regulation of down-stream flowering genes we developed truncation mutants for the A and B genome copies of *VRN1* in tetraploid wheat (henceforth, Δ*vrn-A1* and Δ*vrn-B1*) and combined them to generate two sets of mutants with no functional copies of *VRN1* (henceforth, Δ*vrn1*-null). Using these mutants we demonstrate that functional VRN1 proteins are not essential for wheat flowering or for the up-regulation of *FT*. We also show that *VRN1* expression in the leaves is important for maintaining low transcript levels of *VRN2* in the leaves after vernalization, which is critical for a timely flowering in the spring.

## Results

### Generation of *VRN1* and *VRN2* mutant combinations

#### Screening

The Kronos tetraploid cultivar used to develop the TILLING population carries a functional *VRN-A1* allele, which is defined by a large deletion in intron one associated with spring growth habit. Kronos *VRN-B1* allele has no intron one deletion and is responsive to vernalization [7]. As described in the introduction, the two closely related *ZCCT-B2* genes (*VRN-B2* locus) are the only functional *ZCCT* copies in Kronos [11]. Since the *VRN-A1* allele for spring growth habit is dominant and epistatic to *VRN2*, Kronos has a spring growth habit. This genotype will be referred to as “wild type” in this study.

The screening of the tetraploid Kronos TILLING population [34] with primers described in Table S1 yielded 33 mutations for the *VRN-A1* gene (Δ*vrn-A1*) and 44 mutations for the *VRN-B1* gene (Δ*vrn-B1*). Mutations resulting in truncations or amino acid changes are described in Table S2. Two mutations resulting in either a premature stop codon or the removal of a splice site were selected for each *VRN1* homeolog (Figure S1A). For each of these mutations we recovered homozygous lines, which all show a single chromatogram peak at the mutant site (Figure S1B). This result demonstrates that Kronos has a single copy of each of the *VRN1* homoeologs.

#### Selected mutations

Mutants Δ*vrn-A1_2235_* and Δ*vrn-B1_1254_* have premature stop codons in exons three and four. These mutant alleles encode VRN1 proteins that lack approximately half of the conserved K-box (45% to 66%, respectively) and the complete activation domain (Figure S1A). Sequencing of the Δ*vrn-A1*_2268_ transcripts revealed that the mutation at the acceptor splice site of intron two (AG to AA) resulted in the utilization of the mutated base and the adjacent G in exon three as a new acceptor splice site. This 1-bp frame shift mutation resulted in an altered protein sequence with a premature stop after the first nine amino acids. The mutations at the donor splice site of intron one (GT to AT) in Δ*vrn-B1*_2619_ resulted in an immediate stop codon. Transcripts from this *VRN-B1* allele were not detected in the expression experiments (PCR primers in exon junction 5–6 and exon 8 [5]), likely due to the long (∼10 kb) un-spliced intron one. Proteins encoded by the two splice site mutants are predicted to have truncations including 84% and 100% of the conserved K-box and the complete activation domain. In summary, the large truncations predicted in the four VRN1 mutant proteins are almost certain to knock-out its normal function.

#### Combination of different mutations

The individual mutants were backcrossed once to the non-mutagenized Kronos to reduce background mutations and were then inter-crossed to produce two independent mutants with no functional copies of *VRN1* (Δ*vrn1-*null). The Δ*vrn1-*null set 1 was derived from the stop codon mutants Δ*vrn-A1_2235_* and Δ*vrn-B1_1254_*, and the Δ*vrn1-*null set 2 from the splice site mutants Δ*vrn-A1*_2268_ and Δ*vrn-B1*_2619_ (Figure S1).

The *VRN1* mutations from the Δ*vrn1-*null set 2 were also combined with mutations in the *VRN2* genes from a Δ*vrn2-*null tetraploid mutant identified in a previous study [11]. The Δ*vrn2-*null line carries a deletion encompassing all *ZCCT* genes at the *VRN-B2* locus in addition to the natural mutations present in most durum varieties that cause non-functional amino acids substitutions at conserved positions of the CCT domains of the *ZCCT-A1* and *ZCCT-A2* genes (*VRN-A2* locus) [11]. The Δ*vrn2-*null mutant was backcrossed twice to Kronos and then inter-crossed with the Δ*vrn1-*null to generate a line lacking functional copies of both *VRN1* and *VRN2* genes, which is designated hereafter as Δ*vrn1-*Δ*vrn2-*null. A sister line carrying the functional vernalization-insensitive *VRN-A1* allele (Δ*vrnB1-*Δ*vrn2-*null, spring growth habit) was also selected to be used as control in the vernalization experiments including the Δ*vrn1-*Δ*vrn2-*null mutants. Table 1 summarizes the vernalization responses and the *VRN1* and *VRN2* functional and non-functional alleles present in each mutant.

**Table 1:** Description of *VRN1* and *VRN2* mutants.

Genotype	Vernalization^1^	*VRN1* (flowering promoter)^2^		*VRN2* (flowering repressor)	
		Functional	Non-functional	Functional	Non-functional
Spring growth habit					
Wild type (wt)	**Insensitive**	***VRN-A1*** * VRN-B1*	None	*VRN-B2*	*vrn-A2*
Δ*vrn-B1*	**Insensitive**	***VRN-A1***	*vrn-B1*	*VRN-B2*	*vrn-A2*
Δ*vrn-B1-*Δ*vrn2-null*	**Insensitive**	***VRN-A1***	*vrn-B1*	None	*vrn-A2 vrn-B2*
Winter growth habit					
Δ*vrn-A1*	Sensitive	*VRN-B1*	*vrn-A1*	*VRN-B2*	*vrn-A2*
Δ*vrn1-null*	Sensitive	None	*vrn-A1 vrn-B1*	*VRN-B2*	*vrn-A2*
Δ*vrn1-*Δ*vrn2-null*	Sensitive	None	*vrn-A1 vrn-B1*	None	*vrn-A2 vrn-B2*

1 Vernalization accelerates flowering in the vernalization sensitive genotypes but has no effect in the vernalization insensitive ones.

2 The Kronos line used for mutagenesis carries a vernalization insensitive *VRN-A1* allele with a large deletion in the first intron and a vernalization responsive *VRN-B1* allele (wild type). The presence of the vernalization insensitive *VRN-A1* allele results in a spring growth habit independently of the allele present at the *VRN-B1* locus [7].

### Effects of *VRN1* mutations on heading time and flower morphology

#### Single gene mutants

**Figure 2:**
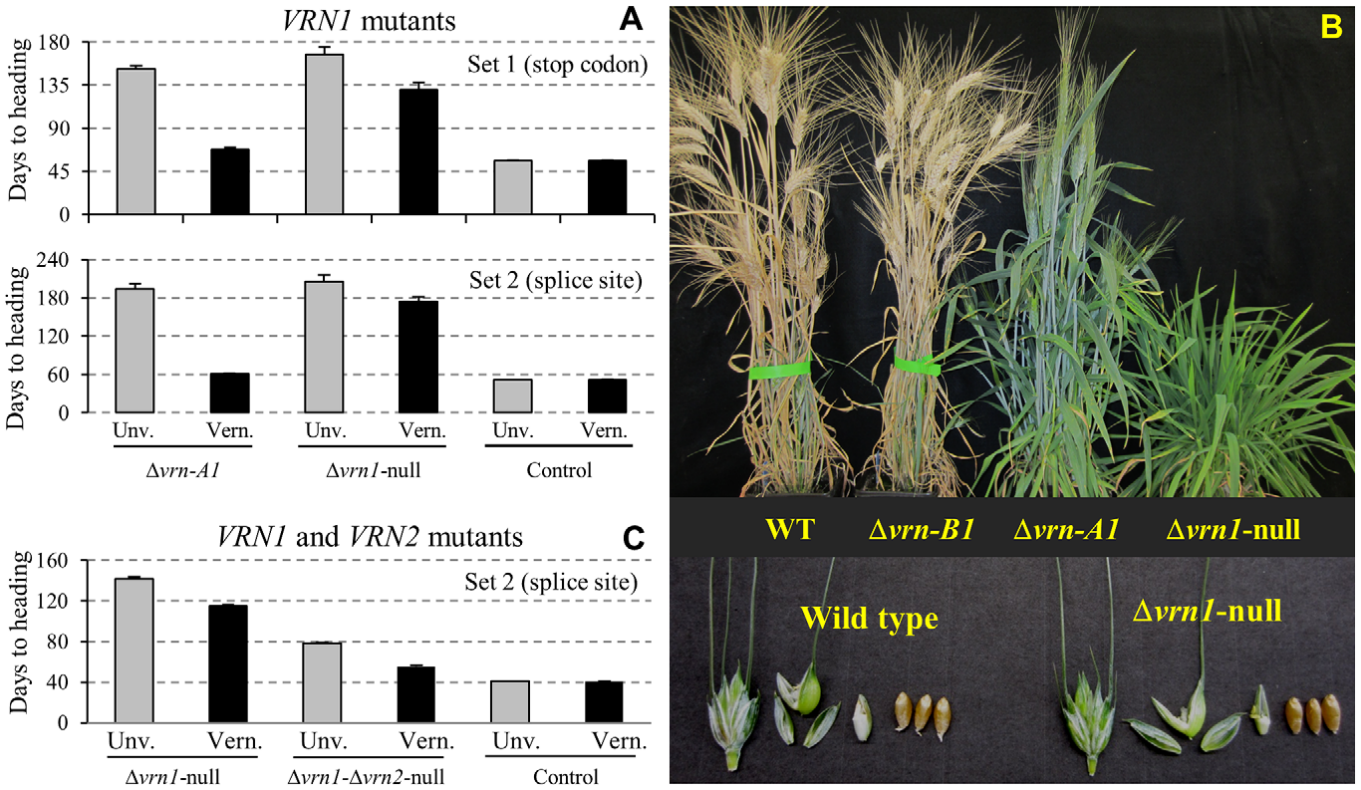
Heading times and spikelet morphology of *VRN1* mutants. Δ*vrn-A1*: mutation in *VRN-A1* (winter, functional vernalization responsive *VRN-B1* allele, functional *VRN2*), Δ*vrn-B1*: mutation in *VRN-B1* (spring, functional vernalization insensitive *VRN-A1* allele, functional *VRN2*), Δ*vrn1-*null: truncated VRN1 proteins (functional *VRN2,* winter), Δ*vrn1-*Δ*vrn2-*null: no functional VRN1 and VRN2 proteins (early). *A*) Growth chamber experiment: Heading times of unvernalized and 6-weeks vernalized Δ*vrn-A1* and Δ*vrn1-*null mutants set 1 (premature stop codon, top) and 2 (splice site mutant, bottom). Control = Δ*vrn-B1* (functional vernalization-insensitive *Vrn-A1* allele). *B*) Top: maturity differences between wild type, single and null *VRN1* mutants (set1). Bottom: spikelet morphology at anthesis and mature seeds from wild type and Δ*vrn1-*null mutants. *C*) Greenhouse experiment comparing heading times of unvernalized and 8-weeks vernalized Δ*vrn1-*null and Δ*vrn1-*Δ*vrn2-*null mutants. These sib lines have the same *VRN1* mutations but differ in the presence or absence of functional *VRN2* genes. Control = Δ*vrn-B1-*Δ*vrn2-*null (functional vernalization-insensitive *VRN-A1* allele and no functional *VRN2* genes). Heading times of the vernalized lines (black bars) are adjusted using the difference in flowering time between vernalized and unvernalized spring control lines as described in Material and Methods.

The unvernalized Δ*vrn-B1* mutants (Δ*vrn-B1* set 1 and Δ*vrn-B1* set 2) flowered as early as the Kronos wild type plants (*P* = 0.38, 51–56 d, Figure 2B). These mutants have a functional *VRN-A1* gene with a large deletion in intron one, which results in its expression to high levels even in the absence of vernalization [7]. These lines are insensitive to vernalization and were used as controls to adjust heading times of the vernalized plants in their respective mutant sets in Figure 2A (black columns, see Material and Methods).

The unvernalized Δ*vrn-A1* mutants flowered 96 (Δ*vrn-A1* set 1) and 143 (Δ*vrn-A1* set 2) days later than the Δ*vrn-B1* mutants (Figure 2A). This is expected since these lines have a functional *VRN-B1* allele that is known to be responsive to vernalization [7]. Vernalization accelerated heading time of both Δ*vrn-A1*_2235_ and Δ*vrn-A1*_2268_ by 84 and 133 days respectively (Figure 2A). These results confirmed that the spring growth habit of Kronos is determined by the *VRN-A1* allele and that the *VRN-B1* allele is responsive to vernalization.

#### *Δvrn1*-null mutants

In the absence of vernalization, the two sets of Δ*vrn1-*null mutants headed 11–15 days later than the Δ*vrn-A1* single mutants but the differences were not significant (Figure 2A, *P*>0.05). However, when these plants were vernalized, the Δ*vrn1-*null mutants flowered 62 and 113 days later than the corresponding Δ*vrn-A1* mutants (Figure 2A, *P*<0.0001), which indicates that the lack of functional VRN1 proteins in the Δ*vrn1-*null mutants greatly reduced their ability to respond to vernalization. This response, however, was not completely eliminated, since vernalized Δ*vrn1-*null mutants still flowered 37 days (*P* = 0.005, set 1) and 31 days (*P* = 0.035, set 2) earlier than the unvernalized Δ*vrn1-*null mutants (Figure 2A). This result was further confirmed in an independent experiment, in which the vernalized Δ*vrn1-*null mutants flowered 27 days earlier than the unvernalized ones (set 2, Figure 2C).

Both vernalized and unvernalized Δ*vrn1-*null mutants produced normal flowers and fertile seeds (Figure 2B, bottom panel), which indicates that *VRN1* is not essential for the initiation of flowering or for floral and seed development in wheat. These results also indicate that there are redundant flowering genes with functions overlapping those of *VRN1*.

#### *Δvrn1-*Δ*vrn2–*null mutants

To determine if the extended delay in flowering of the Δ*vrn1-*null mutants was mediated by the flowering repressor *VRN2*, we compared heading times of the unvernalized sib lines Δ*vrn1-*null (set 2) and Δ*vrn1-*Δ*vrn2-*null. These two lines carry the same *VRN1* splice site mutations but differ in the presence or absence of functional *VRN2* genes. On average, the unvernalized Δ*vrn1-*Δ*vrn2-*null plants headed 63 days earlier than the unvernalized Δ*vrn1-*null sib lines (Figure 2C), which indicates that the *VRN2* gene has a large effect on the late flowering phenotype of the Δ*vrn1-*null mutants.

Even though the unvernalized Δ*vrn1-*Δ*vrn2-*null flowered 2 months earlier than the Δ*vrn1-*null mutants, they still flowered 37 days later than the unvernalized Δ*vrn-B1*-Δ*vrn2-*null control that carries a functional vernalization-insensitive *VRN-A1* allele (Table 1). Since these two mutants differ only in the presence or absence of the *VRN-A1* allele, it can be concluded that this allele has the ability to accelerate flowering even in the absence of *VRN2* (Figure 2C).

A comparison between the un-vernalized and vernalized Δ*vrn1-*Δ*vrn2-*null plants showed that the vernalized plants flowered on average 23 days earlier than the unvernalized ones (Figure 2C, *P*<0.0001). This result indicates that the redundant genes responsible for flowering in the absence of functional VRN1 proteins are able to respond to vernalization even in the absence of VRN2.

### Effect of the *VRN1* mutations on transcript levels of *VRN2* and *FT*

To understand better the large differences in flowering time observed in the *VRN1* and *VRN2* mutants we studied the effect of these mutations on the transcription profiles of *VRN1, FT* and *VRN2* by quantitative RT-PCR (qRT-PCR). Since *FT* and *VRN1* transcription profiles are similar, they are described together.

#### *FT* and *VRN1*

**Figure 3:**
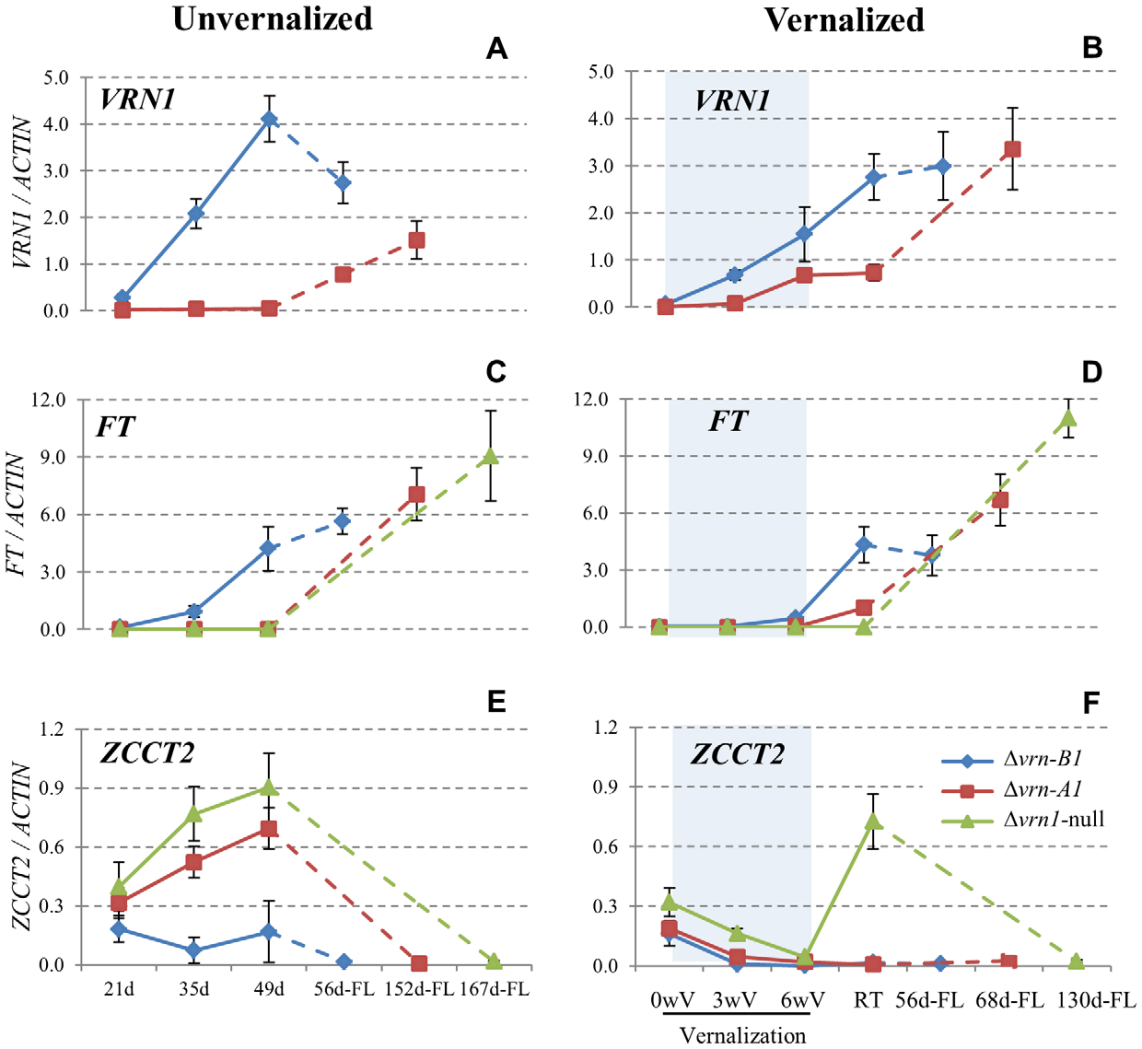
qRT–PCR transcriptional profiles of *VRN1*, *FT*, and *ZCCT2* ( = *VRN2*) in mutant set 1 (premature stop codon). A–B) *VRN1*, C–D) *FT,* E–F) *ZCCT2*. Left panels A, C and E) unvernalized plants. Right panels B, D, and F) vernalized plants. Δ*vrn-A1*: mutation in *VRN-A1* (winter, functional vernalization responsive *VRN-B1* allele), Δ*vrn-B1*: mutation in *VRN-B1* (spring, functional vernalization insensitive *VRN-A1* allele), Δ*vrn1-*null: truncated VRN1 proteins. Blue shaded areas indicate vernalization at 4°C under long days. 0 wV: 3 weeks-old plants grown under 22°C/17°C (day/night) conditions before vernalization, 3 wV: 3 weeks of vernalization, 6 wV: 6 weeks of vernalization, RT: two weeks after returning the vernalized plants to pre-vernalization conditions. A final sample was obtained from flag leaves at heading times (FL, dotted lines), which are indicated in days from sowing to heading. The X axis scale is not proportional to time and the Y scale is in fold-*ACTIN* values. Error bars are SE of the means from 8 biological replications.

In both unvernalized and vernalized plants, *VRN1* (Figure 3A–3B) and *FT* (Figure 3C–3D) transcript levels were up-regulated earlier during development in the spring Δ*vrn-B1* mutants (blue lines) than in the other genotypes, in agreement with their early heading time. No significant differences in the expression profiles of these genes between Kronos and the Δ*vrn-B1* mutants were detected, which is consistent with the very similar heading times of these lines (data not shown). The winter Δ*vrn-A1* mutants (red lines) showed a significant up-regulation of *VRN1* during vernalization (Figure 3B) as expected for a line carrying a functional vernalization-responsive *VRN-B1* allele.

In the Δ*vrn1-*null mutants, transcript levels of the mutant *VRN1* alleles increased significantly by the end of vernalization (*P* = 0.01). However, since it is not possible to separate the effect of the truncated VRN1 proteins on its transcriptional regulation from the effects of the mutations on RNA stability, the *VRN1* transcript profiles in the Δ*vrn1-*null mutants were excluded from the figures and were not discussed further. The Δ*vrn1-*null mutants took a long time to flower, but at heading time the transcript levels of *FT* in the flag leaves were high in both vernalized and unvernalized plants (Figure 3, dotted lines). Almost identical transcription profiles were observed in the second set of *VRN1* mutants (Figure S2A–S2D) confirming the previous results.

#### *VRN2*

In the unvernalized plants, an inverse correlation was observed between the transcript levels of *VRN1* and *VRN2*, similar to the one described in previous studies [22]. The Δ*vrn-B1* spring mutants (Figure 3E, blue solid lines) showed the highest transcript levels of *VRN1* and the lowest transcript levels of *VRN2* and the opposite was observed in the Δ*vrn-A1* and Δ*vrn1-*null winter mutants (Figure 3E, red and green solid lines).

The comparison of the *VRN2* transcript profiles among the different genotypes revealed two important results. First, the *VRN2* genes were down-regulated during vernalization in both the Δ*vrn1-*null and Δ*vrn-A1* mutants (Figure 3F, *P* = 0.002, and Figure S2F, *P* = 0.0009). This result shows for the first time that *VRN1* is not essential for the down-regulation of *VRN2* by vernalization. Second, a rapid increase in the transcript levels of *VRN2* to pre-vernalization levels was observed two weeks after the vernalized plants were returned to room temperature in the Δ*vrn1-*null mutants but not in the other genotypes (Figure 3F, RT). At the same time point, the Δ*vrn1-*null mutants showed lower transcript levels of *FT* (Figure 3D, RT) and *VRN1* (Figure 3B, RT) than the Δ*vrn-A1* mutants. Similar results were confirmed in the second set of Δ*vrn1-*null mutants (Figure S2F).

We conclude from these results that an important role of *VRN1* in the leaves is to maintain the repression of the *VRN2* genes after vernalization. The inability of the Δ*vrn1-*null mutants to maintain low levels of *VRN2* expression after vernalization correlates well with the reduced vernalization response of the Δ*vrn1-*null mutants relative to the Δ*vrn-A1* mutants (Figure 2A).

Even though the Δ*vrn1-*null mutants showed a large delay in flowering time they eventually flowered. At heading time the transcript levels of *VRN2* in the flag leaves were almost undetectable (Figure 3E–3F and Figure S2E–S2F) and the transcript levels of *VRN1* and *FT* were relatively high (Figure 3A–3D and Figure S2A–S2D dotted lines), both in the vernalized and unvernalized plants. These results indicate that *VRN2* can be down-regulated during development independently of *VRN1.*

### Effect of the *VRN2* mutations on *FT* transcript levels

**Figure 4:**
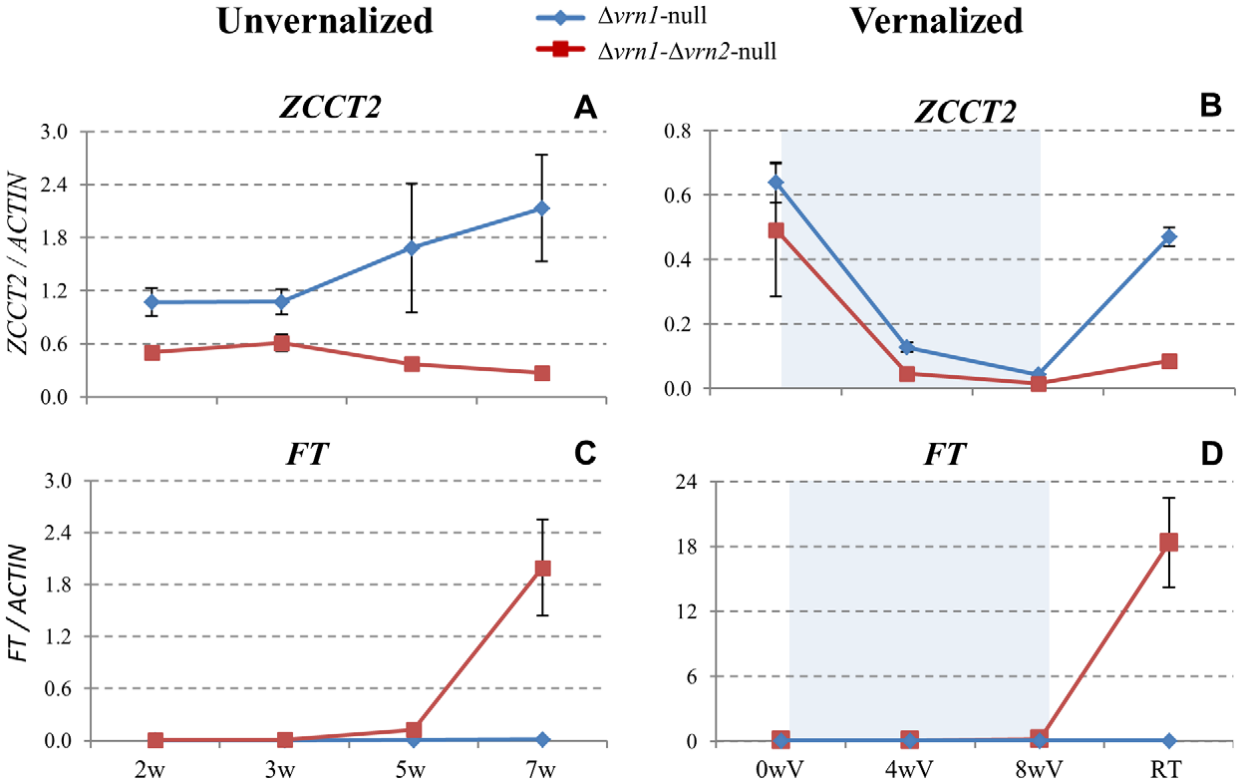
Comparison of *VRN2* and *FT* transcript levels between Δ*vrn1-*null and Δ*vrn1-*Δ*vrn2-*null mutants. A–B) *ZCCT2,* C–D) *FT.* Left panels A and C) unvernalized plants. Right panels B and D) vernalized plants. Δ*vrn1-*null: no functional VRN1 proteins (functional *VRN2,* winter), Δ*vrn1-*Δ*vrn2-*null: truncated VRN1 and VRN2 proteins (early). Blue shaded areas indicate vernalization at 4°C under long days. 0 wV: 3 weeks-old plants grown under 22°C/17°C (day/night) conditions before vernalization, 4 wV: 4 weeks of vernalization, 8 wV: 8 weeks of vernalization, RT: two weeks after returning the vernalized plants to pre-vernalization conditions. A–B) ZCCT2 ( = *VRN2*). Lower transcript levels in the Δ*vrn1-*Δ*vrn2-*null mutant (red line) are likely caused by the complete deletion of the *VRN-B2* genes in this line. Only the non-functional *ZCCT-A2* transcripts are detected, C–D = *FT.* Note the rapid up-regulation of *FT* in the Δ*vrn1-*Δ*vrn2-*null mutants relative to the Δ*vrn1-*null mutants. The X axis scale is in weeks (w) and is not proportional to time. The Y scale is in fold-*ACTIN* values. Error bars are SE of the means from 8 biological replications.

To study the role of *VRN2* in the absence of functional VRN1 proteins, we compared the transcriptional profiles of the Δ*vrn1-*null and Δ*vrn1-*Δ*vrn2-*null mutants. These two mutants have the same *VRN1* mutations but differ in the presence or absence of functional *VRN2* genes (Figure 4). Since these two mutants lack any functional *VRN1* genes, only the transcript profiles of *VRN2* (*ZCCT2*) and *FT* are presented in Figure 4.

#### *VRN2* (*ZCCT2*)

The Δ*vrn1-*null mutants showed higher levels of *ZCCT2* transcripts than the Δ*vrn1-*Δ*vrn2-*null mutants (Figure 4A). This is expected since the Δ*vrn1-*Δ*vrn2-*null mutants have a deletion encompassing the *ZCCT-B2* genes, and only the transcripts of the non-functional *ZCCT-A2* gene are detected in this line. The down-regulation of *ZCCT2* during vernalization was similar in both genotypes. However, when plants were returned to room temperature the up-regulation of *ZCCT2* was higher in the Δ*vrn1-*null than in the Δ*vrn1-*Δ*vrn2-*null mutants (Figure 4B).

#### *FT*

The Δ*vrn1-*null mutants (functional *VRN2*) showed undetectable levels of FT both in the vernalized and the un-vernalized plants during this experiment. In contrast, the Δ*vrn1-*Δ*vrn2-*null mutants (non-functional *VRN2*) showed very high transcript levels of *FT* by the end of the experiment, both in the vernalized and unvernalized plants (Figure 4C–4D). The significantly higher levels of *FT* observed in the Δ*vrn1-*Δ*vrn2-*null than in the Δ*vrn1-*null mutants correlate well with the two-month flowering difference observed between these two mutants (Figure 2C). These results also demonstrate that the ability of *VRN2* to repress *FT* transcription is not dependent on *VRN1*.

Interestingly, *FT* transcript levels in the Δ*vrn1-*Δ*vrn2-*null mutants jumped from undetectable levels to 18-fold *ACTIN* two weeks after the plants were removed from vernalization. In the unvernalized plants, *FT* increased only from 0.1 to 1.8-fold *ACTIN* during the last two weeks of the experiment. This result is consistent with the significant acceleration in flowering time observed in the Δ*vrn1-*Δ*vrn2-*null mutants (23 days) after eight weeks of vernalization.

### Preliminary characterization of *VRN1*'s closest paralogs *FUL2* and *FUL3*

Even in the absence of functional VRN1 proteins, vernalization accelerated flowering of the Δ*vrn1-*null and Δ*vrn1-*Δ*vrn2-*null mutants (Figure 2C), which indicates the existence of additional vernalization responsive genes. To test if *VRN1*'s closest paralogs *FUL2* and *FUL3* were responsive to vernalization, we characterized their expression profiles using the same cDNA samples obtained from the vernalization experiments described in Figure 3.

#### *FUL2* and *FUL3* are expressed at high levels in the leaves

In the leaves of unvernalized *VRN1* mutants, *FUL2* and *FUL3* transcripts levels increased during development reaching high levels in the flag leaves (>6-fold *ACTIN,* Figure S3A and S3C), that were even higher than those described previously for *VRN1* (Figure 3). Similar to *VRN1* (Figure 3), the up-regulation of *FUL2* and *FUL3* transcripts levels occurred earlier in development in the spring Δ*vrn-B1* mutants than in the winter Δ*vrn-A1* and Δ*vrn1-*null mutants (Figure S3).

#### *FUL2* and *FUL3* transcript levels are up-regulated by vernalization independently of *VRN1*

**Figure 5:**
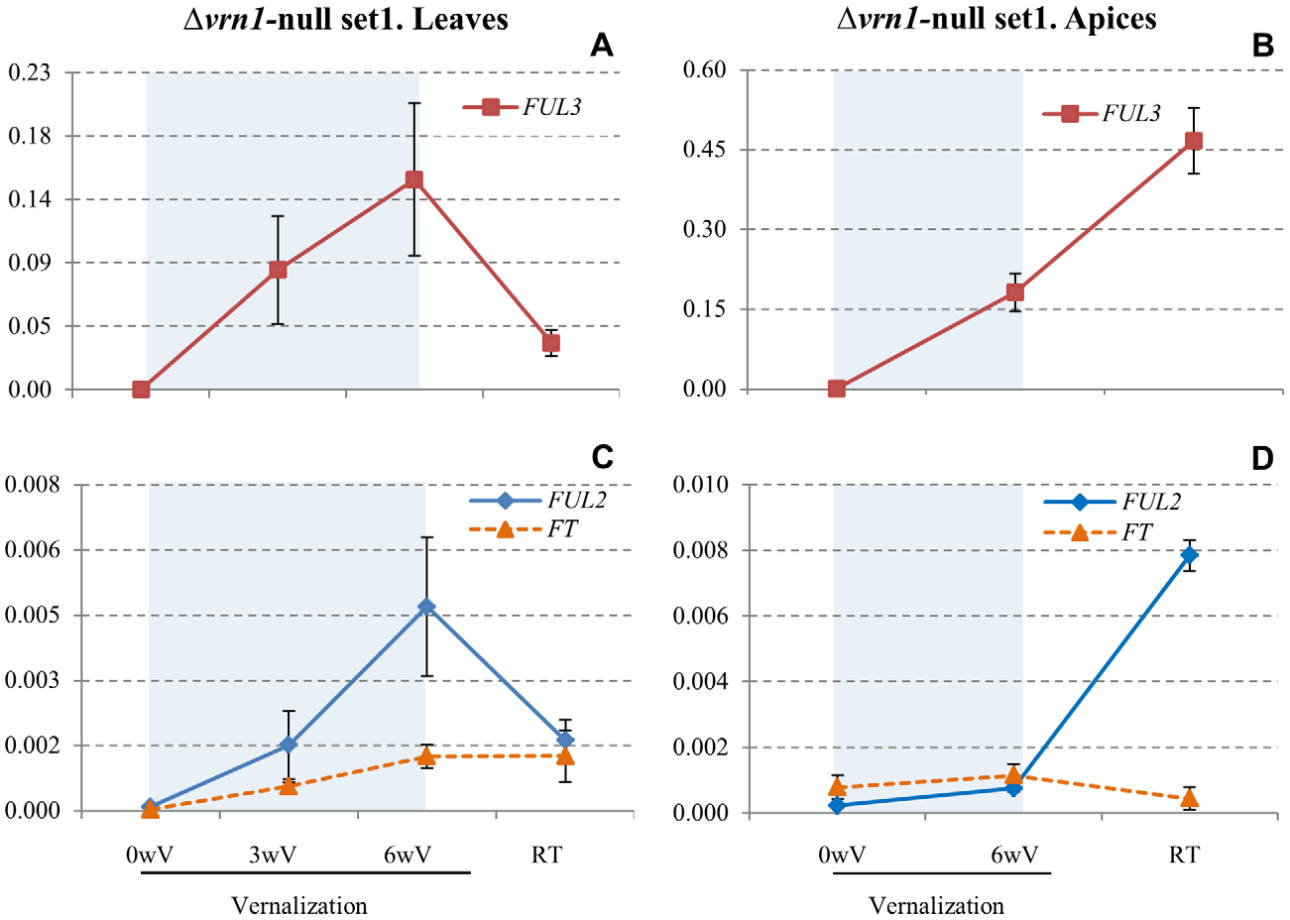
Transcriptional profiles of *FUL2, FUL3*, and *FT* during and after vernalization in the Δ*vrn1-*null mutant set 1 (premature stop codon). A–B) *FUL3*, C–D) *FUL2*, and *FT.* Left panels A and C) samples from leaves. Right panels B and D) samples from apical region. The blue shaded area indicates vernalization at 4°C under long days. 0 wV: 3 weeks-old plants grown at 22°C/17°C (day/night) immediately before vernalization, 3 wV: 3 weeks of vernalization, 6 wV: 6 weeks of vernalization, RT: two weeks after removing the plants from the cold and returning them to pre-vernalization conditions. The X axis scale is not proportional to time and the Y scale is in fold-*ACTIN* values. Error bars are SE of the means from 8 biological replications in the leaves and 3 biological replications in the apices (each including a pool of 30 shoot apical meristem and surrounding tissue).

*FUL2* and *FUL3* transcript levels were significantly (*P*<0.01) up-regulated by vernalization in the leaves of the Δ*vrn1-*null mutants, both in set 1 (Figure 5A–5D) and set 2 (Figure S4A–S4B). After six weeks of vernalization, *FUL3* transcript levels in the Δ*vrn1-*null mutants reached levels similar to those observed for *VRN1* (10–15% *ACTIN* level), but *FUL2* transcript levels were more than 20-fold lower (0.5% *ACTIN* level). As previously reported for *VRN1* [1], the increase of the transcript levels of *FUL2* and *FUL3* was proportional to the duration of the vernalization treatment (Figure 5A and 5C, Figure S4A–S4B). These results indicate that *FUL2* and *FUL3* can be up-regulated by vernalization in the absence of functional VRN1 proteins.

Interestingly, when Δ*vrn1-*null mutant plants were removed from the cold and allowed to recover at room temperature for two weeks, transcript levels of *FUL2* and *FUL3* returned to pre-vernalization levels in the mature leaves (Figure 5A and 5C, Figure S4A–S4B) but continued to increase in the actively dividing apices (Figure 5B and 5D). This up-regulation in the apices is not associated with changes in *FT* (Figure 5C–5D) which is maintained at low levels in the leaves due to the up-regulation of *VRN2* (*FT* repressor) in the Δ*vrn1-*null mutant (Figure 3F).

#### *FUL2* and *FUL3* are negatively regulated by *VRN2*

In contrasts with the Δ*vrn1-*null mutants, the transcript levels of *FUL2* and *FUL3* in the leaves of the Δ*vrn1-*Δ*vrn2-*null mutants, increased to very high levels (>10-fold *ACTIN,* Figure S5A–S5B) when the vernalized plants were returned to room temperature. Since these two sister mutant lines differ only in the presence of functional *VRN2* genes, these results indicate that *VRN2* has a negative effect on the transcriptional regulation of *FUL2* and *FUL3* in the leaves. As described below, this effect is likely mediated by the negative effect of *VRN2* on *FT* transcription.

#### *FUL2* and *FUL3* are positively regulated by *FT*

The elimination of all functional copies of *VRN2* in the Δ*vrn1-*Δ*vrn2-*null mutants resulted in a significant up-regulation of *FT* in the leaves after vernalization relative to the Δ*vrn1-*null mutants (Figure 4D), which suggested the possibility that the negative effect of *VRN2* on *FUL2* and *FUL3* was mediated by *FT*. To test this hypothesis, we analysed the transcript levels of *FUL2* and *FUL3* in two pairs of isogenic lines differing in *FT* transcript levels and heading time [5] (Figure S6A).

The first pair of isogenic lines included the late-spring variety Chinese Spring (CS) and a substitution line of chromosome 7B from the variety Hope in CS (henceforth, CS-H7B). The Hope 7B chromosome carries an *FT* allele with an insertion of a repetitive element in its promoter that is associated with high transcript levels of *FT* [5]. The second pair of isogenic lines included the winter wheat variety Jagger (JAG) and transgenic Jagger plants (JAG-OE) transformed with the *FT* allele from Hope [5]. The transgenic JAG-OE and the chromosome substitution line CS-H7B flowered significantly earlier (*P*<0.0001) than their respective controls (Figure S6A).

After 7 weeks at room temperature, *FT* transcript levels remained undetectable in the lines carrying the wild type *FT* alleles (CS and JAG), but were 7 and 21-fold higher than *ACTIN* in CS-H7B and JAG-OE, respectively (Figure S6B). The higher levels of *FT* in CS-H7B and JAG-OE were associated with significantly higher transcript levels of *FUL2* and *FUL3* relative to the control lines (Figure S6B and S6C), which suggests that these two genes are positively regulated by *FT*.

## Discussion

### *VRN1* is not essential for wheat flowering

The dramatic non-flowering phenotype of the wheat *mvp* mutants suggested initially that *VRN1* was an essential flowering gene [33]. However, a later study showed that the deletions in the *mvp* mutants including *VRN1* were larger than initially proposed, and encompassed several genes including *PHYC*, an important light receptor and *AGLG1,* the wheat ortholog of rice *PAP2* [14]. Phytochromes affect flowering time in Arabidopsis [35] and rice [36] and *PAP2* affects flowering time and reproductive development in rice [32] and therefore cannot be ruled out as an alternative cause of the non-flowering phenotype of the *mvp* mutants.

The Δ*vrn1-*null mutants developed in this study allowed us to separate the effect of *VRN1* from the effect of the other genes included in the *mvp* deletions. The production of normal flowers and seeds in the Δ*vrn1-*null and Δ*vrn1-vrn2-*null mutants demonstrates that *VRN1* is not essential for wheat flowering, and that the non-flowering phenotype of the *mvp* mutants is not solely determined by the deletion of *VRN1.* In addition, the early flowering time of the Δ*vrn1-vrn2-*null mutant indicates the existence of redundant flowering genes that are capable of rapidly inducing flowering in wheat in the absence of functional VRN1 and VRN2 proteins.

### Even in the absence of *VRN1* there is a significant vernalization response

Vernalization accelerated flowering time by 84–133 days in the two Δ*vrn-A1* mutants (functional vernalization responsive *VRN-B1* allele), but only by 31–37 days in the Δ*vrn1-*null mutants relative to the unvernalized controls. This three- to four- fold reduction in the acceleration of flowering by vernalization in the mutants with truncated copies of *VRN1* confirms the important role this gene plays in the vernalization response in wheat. However, the fact that a significant acceleration of flowering by vernalization was still detected in the Δ*vrn1-*null mutants indicates the existence of unidentified genes with the ability to respond to vernalization. This significant response to vernalization in the absence of *VRN1* does not seem to be dependent on the presence of *VRN2,* because the Δ*vrn1-vrn2-*null mutants also showed a significant acceleration of flowering by vernalization.

### VRN1 plays a central role in the repression of *VRN2* in the leaves after winter

Loukoianov and collaborators [22] observed a negative correlation between the transcript levels of *VRN1* and the transcript levels of *VRN2* in the leaves of isogenic hexaploid wheat lines carrying different *VRN1* spring alleles, and hypothesized that *VRN1* functions as a negative transcriptional regulator of *VRN2*. This negative interaction became an integral part of a feedback regulatory loop placed at the center of current flowering models (Figure 1), but has never been conclusively demonstrated before this study.

Results from the Δ*vrn1-*null mutants confirmed the validity of the previous hypothesis. Only the mutant lines with no functional copies of *VRN1* showed an up-regulation of *VRN2* transcript to pre-vernalization levels when the plants were removed from the vernalization treatment and were transferred back to room temperature (Figure 3F and Figure S2F). This result, observed in the two independent sets of Δ*vrn1-*null mutants, confirmed that one specific role of *VRN1* is to maintain low transcript levels of *VRN2* in the leaves following winter, when the longer day length and warmer weather conditions favour the induction of *VRN2* transcription. The transcriptional activation of *VRN1* in the leaves during vernalization provides an important regulatory signal that controls the transition from vegetative growth in the fall to reproductive development in the spring. In the fall, low *VRN1* transcript levels result in high *VRN2* expression, the repression of *FT,* and the maintenance of vegetative growth. During the winter *VRN1* is up-regulated and *VRN2* is down-regulated. The presence of *VRN1* in the leaves prevents *VRN2* from being up-regulated during the longer and warmer days of spring, which facilitates the transcriptional activation of *FT* and the induction of flowering. In summary, the negative regulation of *VRN2* by *VRN1* in the leaves is a key regulatory step in the seasonal responses of winter wheat. It remains to be determined whether the VRN1 protein interacts directly with the *VRN2* gene or whether other genes mediate this regulatory interaction.

### *VRN1* is not required for the down-regulation of *VRN2* during vernalization and during development of unvernalized plants

Previous vernalization experiments conducted under short-day conditions demonstrated that *VRN1* transcript levels were up-regulated by vernalization in the absence of detectable levels of *VRN2* and *FT* transcripts [23], [24]. Based on these results it was assumed that vernalization operated mainly on *VRN1* and that the downregulation of *VRN2* during vernalization was likely an indirect effect of the up-regulation of *VRN1* [12], [37]. This hypothesis was reinforced by the observation that vernalization promotes an active chromatin state in *VRN1* regulatory regions but not in those of *VRN2* or *FT* [25].

However, in this study we observed down-regulation of *VRN2* during vernalization in both sets of Δ*vrn1*-null mutants, which encode for truncated VRN1 proteins (Figure 3F and Figure S2F). This result demonstrates that the down-regulation of *VRN2* during vernalization does not require the presence of *VRN1.*

Similarly, a developmental down-regulation of *VRN2* was observed in both sets of unvernalized Δ*vrn1-*null plants (Figure 3E and Figure S2E, dotted lines). This result suggests that, in addition to *VRN1*, there are other negative regulators of *VRN2* that are developmentally regulated.

We are currently developing a triple *vrn1*-*ful2*-*ful3* mutant to test if the induction of *FUL2* and/or *FUL3* transcripts during vernalization (Figure 5 and Figure S4A–S4B) and development (Figure S3A and S3C) play a role in the down-regulation of *VRN2.*

### Results from the *VRN1* mutants shed light on conflicting flowering models

In rice, the simultaneous down-regulation of closely related *FT* paralogs *Hd3a* and *RFT1* by RNAi results in non-flowering plants [38]. Therefore, it is tempting to speculate that the permanent down-regulation of *FT* in the *mvp* mutants could account for their non-flowering phenotype.

**Figure 6:**
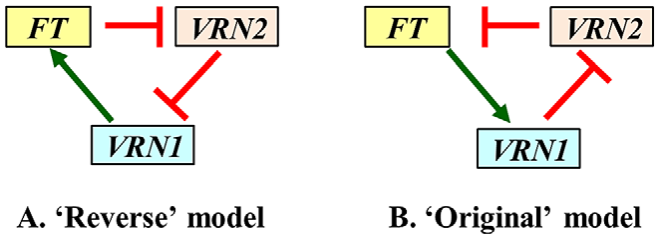
Alternative flowering models in the temperate grasses. A) ‘Reverse model’ [13], B) ‘Original model’ [12], [37].Green arrows indicate promotion of transcription and red lines repression of transcription.

To explain the low levels of *FT* transcripts detected in the *mvp* mutants, Shimada and collaborators (2009) proposed an alternative flowering model for the temperate cereals in which *VRN1* promotes *FT* transcription independently of *VRN2* [13] (Figure 6A). In this model, the low *FT* transcript levels of the *mvp* mutants are caused by the *VRN1* deletion. This model also proposes that FT acts as a transcriptional repressor of *VRN2,* and VRN2 represses *VRN1* [13] (Figure 6A). Shimada's model includes a regulatory feedback loop including the same three genes present in the model in Figure 1 but operating in the opposite direction, and will be referred hereafter as the ‘reverse’ model (Figure 6A). The simplified model from Figure 1, included for comparison in Figure 6B, will be referred to as the ‘original’ model for the following discussion.

The reverse model assumes that the deletion of *VRN1* is the cause of the low transcript levels of *FT* in the *mvp* mutants. However, our results indicate that *FT* can reach very high transcript levels in the absence of functional VRN1 proteins both in the Δ*vrn1-*null and Δ*vrn1-vrn2-*null mutants (Figure 3C–3D, Figure S2C–S2D, Figure S5B). Although, we cannot completely rule out the very unlikely possibility that the truncated VRN1 proteins in the Δ*vrn1-*null mutants are able to up-regulate *FT*, we favor the hypothesis that the permanent down-regulation of *FT* in the *mvp* mutants is caused by the deletion of additional genes closely linked to *VRN1* and included in the same deletion. The *PHYC* is a potential candidate to explain these differences since mutations in the *PHYC* gene are known to affect flowering time in both rice [36] and Arabidopsis [35]. However, the effects described in these model species are not as large as those observed in the *mvp* mutants, suggesting the possibility that the non-flowering phenotype of the *mvp* mutants is caused by other genes in the deletion or by the combination of deletions of more than one gene. We are currently developing a wheat *phyC-*null mutant to test the contribution of this gene to the regulation of flowering time in wheat. Another potential candidate among the genes deleted in the *mvp* mutants is the MADS-box gene *AGLG1.* The rice homolog of *AGLG1* (*PAP2*) is expressed in the shoot apical meristem during the initiation of the reproductive stage at the same time as the meristem identity genes (*OsMADS14* = *VRN1*, *OsMADS15* = *FUL2* and *OsMADS18* = *FUL3*). A null allele of the *PAP2* locus combined with reduced expression of the three meristem identity genes by RNA interference down-regulates *FT* expression in the leaves and inhibits the transition of the apical meristem from the vegetative to reproductive phase [32]. In wheat, *AGLG1* transcripts are detected in the developing spike later than *VRN1* and are not detected in the leaves [1]. These results suggest that these homologous genes play different roles in the regulation of flowering initiation in rice and wheat.

The transcription profiles of *FT* and *VRN2* in the Δ*vrn1-*null and Δ*vrn1-vrn2-*null mutants provide additional evidence that contradicts the predictions of the ‘reverse’ flowering model. This comparison shows that even in the absence of functional copies of *VRN1*, the deletion of *VRN2* results in the up-regulation of *FT* (Figure 4C–4D). This result is difficult to explain by the reverse model, which proposes that the negative regulation of *FT* by VRN2 requires the presence of functional VRN1 proteins (Figure 6A).

In addition to the genetic evidence discussed above, biochemical interactions described in previous studies support the interactions proposed in the ‘original’ model. The proposed regulation of *VRN1* by FT (Figure 6B) in wheat is consistent with the known interactions between FT and *AP1* (*VRN1* homolog) in Arabidopsis. In this species, the FT protein is synthesized in the leaves and is transported through the phloem to the stem apical meristem where it forms a complex with the bZIP transcription factor FD and binds to the promoter of *AP1* [17]. Similarly, the wheat FT protein interacts with the bZIP transcription factor FDL2 which interacts *in vitro* with the *VRN1* promoter [18]. In rice, the FT homolog (Hd3a) interacts with OsFD1 and a 14-3-3 protein to form a complex that binds the promoter of the rice homolog of *FUL2* (*OsMADS15*) [39]. A biochemical mechanism has been proposed also for the repression of *FT* by VRN2 [15]. Using a yeast-three-hybrid system Li et al. (2011) showed that VRN2 and CONSTANS proteins compete for binding with a common set of NF-Y transcription factors, which have been shown to be involved in the regulation of *FT* in Arabidopsis [20], [21]. In summary, available genetic and biochemical evidence support the ‘original’ model of flowering for the temperate cereals [12], [37].

### Future directions

The fact that both sets of Δ*vrn1-*null null mutants were able to flower and set normal seeds, demonstrated the existence of redundant flowering genes with meristem identity functions overlapping those of *VRN1*. The MADS-box genes *FUL2* and *FUL3* are the closest paralogs of *VRN1* [26] and therefore, the most parsimonious hypothesis is that their encoded proteins have retained meristem identity functions that can compensate for the lack of a functional VRN1 protein. A similar redundant system exists in Arabidopsis, in which only simultaneous mutations in the three duplicated meristem identity genes *AP1-CAL–FUL* results in mutants that are unable to form flowers under all tested environmental conditions [31]. The existence of functional redundancy in the meristem identity genes is also evident in rice, where the down-regulation of individual meristem identity genes *OsMADS14* ( = *VRN1*), *OsMADS15* ( = *FUL2*) or *OsMADS18* (*FUL3*) has limited effect on flowering time [32], [40]. Only the simultaneous downregulation of the three rice meristem identity genes in combination with *pap2* mutants resulted in extensive delays in stem elongation and severely perturbed inflorescence development [32].

In wheat and barley, previous studies have provided some indirect evidence that support the hypothesis that FUL2 and FUL3 have retained overlapping functions with VRN1. First, *in situ* hybridization studies have shown similar spatial and temporal transcription profiles of these three genes during the initial stages of spike development [29], [30], [41], [42]. In addition, wheat *FUL2* and rice *FUL3* genes were shown to induce flowering when over expressed in transgenic Arabidopsis and rice plants [30], [40].

The experiments described in this study showed additional similarities between *FUL2, FUL3* and *VRN1* genes. First, the transcript levels of *FUL2* and *FUL3* were significantly up-regulated during vernalization independently of *VRN1* and this up-regulation was proportional to the duration of the cold treatment, as described before for *VRN1* (Figure 5 and Figure S4A–S4B). Second, the transcript levels of *FUL2* and *FUL3* were negatively regulated by *VRN2* (Figure 5 *versus* Figure S5A–S5B) and positively regulated by *FT* in transgenic wheat plants overexpressing *FT* (Figure S6B–S6D). Finally, when Δ*vrn1-*null mutant plants were moved from the cold to room temperature, *FUL2* and *FUL3* transcripts returned to pre-vernalization levels in the mature leaves but not in the actively dividing apices (Figure 5). Since cell division is required to establish epigenetic changes, the different transcriptional profiles in these two tissues may reflect the epigenetic regulation of *FUL2* and *FUL3* by vernalization, as shown before for *VRN1* [25]. Studies of the chromatin changes in the regulatory regions of *FUL2* and *FUL3* will be required to rule out alternative explanations. In summary, the similar patterns of transcriptional regulation, together with the similar *in situ* hybridization profiles in early spike development and the early flowering of *FUL2* and *FUL3* transgenic plants, support the existence of some overlapping functions between *FUL2*, *FUL3* and *VRN1*. We have initiated the development of a triple *vrn1-ful2-ful3* mutant in tetraploid wheat to test the roles of *FUL2* and *FUL3* in flowering initiation in wheat.

In addition to the above similarities, a critical difference was observed in the ability of these three meristem identity genes to regulate *VRN2* expression in the leaves. In the absence of functional *VRN1* genes, *VRN2* was up-regulated after vernalization in the Δ*vrn1-*null mutants, in spite of the presence of functional *FUL2* and *FUL3* genes. This result suggests that *FUL2* and *FUL3* are unable to maintain the repression of *VRN2* after vernalization.

In summary, this study demonstrates that a central role of *VRN1* in the leaves is to maintain the repression of *VRN2* after the winter to promote timely flowering in the spring. In spite of its important role in the seasonal regulation of flowering, our results demonstrate that *VRN1* is not essential for flowering and raise new questions regarding the roles of *FUL2, FUL3, AGLG1* and *PHYC* in the regulation of flowering initiation in wheat. Flowering time is a key component of wheat adaptation and productivity and therefore, a precise understanding of the regulatory mechanisms involved in the initiation of flowering will be beneficial to engineer wheat cultivars better adapted to a changing environment.

## Materials and Methods

### Mutant screen

A TILLING (for Targeting Local Lesions IN Genomes) population of 1,368 lines of the tetraploid wheat cultivar ‘Kronos’ mutagenized with ethyl methane sulphonate (EMS) [34] was screened for mutations in the A and B genome copies of *VRN1* using the genome specific primers described in Table S1. Two *VRN1* genomic regions, one including exon one and the other including exons three to six, were targeted for mutant detection using the *Cel*I assay described before [34]. The *VRN2* natural mutants have been described before [14].

### qRT–PCR

RNA samples were extracted from leaves using the Spectrum Plant Total RNA Kit (Sigma-Aldrich). Purified RNA samples were checked for RNA integrity by running 0.5 µg RNA on a 1% agarose gel. All samples showed clear 18S and 25S ribosomal RNA bands indicating lack of RNA degradation. Melting curves showed a single peak, which confirmed amplification of a single product. Standard curves were constructed to calculate amplification efficiency for the SYBR Green systems developed for *FUL2* and *FUL3* (Table S3). SYBR Green systems for *VRN1, ZCCT2* and *FT* were developed in previous studies [5], [11]. All primers used for qRT-PCR are conserved between the A- and B- genome copies of their respective targets. Quantitative PCR was performed using SYBR Green and a 7500 Fast Real-Time PCR system (Applied Biosystems). *ACTIN* was used as an endogenous control using primers described before [43].

Transcript levels for all genes and experiments presented in this study are expressed as linearized fold-*ACTIN* levels calculated by the formula 2^(*ACTIN* CT – *TARGET* CT)^. The resulting number indicates the ratio between the initial number of molecules of the target gene and the number of molecules of *ACTIN* and therefore, the Y scales are comparable across genes and experiments. Primer efficiencies were all higher than 95% (Table S3) and therefore, were not included in the calculation of the linearized values. In the Δ*vrn1-*null set 2 and the derived Δ*vrn1-vrn2-*null mutants the *VRN-B1* transcripts were not detected in the qRT-PCR experiments.

### Experimental conditions for the different experiments

The flowering experiments for the *VRN1* mutants were performed in CONVIRON growth chambers. Long day photoperiod experiments were performed with 16 h of light (6 am to 10 pm, including 1 hour ramp at the beginning and end of the cycle) and 8 h of dark. Intensity of the sodium halide lights measured at plant head height was 260 µM s^−1^. For the unvernalized plants day temperatures were set at 22°C and night temperatures at 17°C respectively. Relative humidity in the chambers was maintained at 60% throughout the entire experiment.

All the vernalization experiments in this study were performed under long days to separate the effect of low temperature from the overlapping effects of short days. Day and night temperatures were 4°C whereas light intensity and relative humidity were identical to the unvernalized experiments. Unvernalized control plants were planted 5 weeks after the sowing of the vernalized plants to allow both groups of plants to reach a similar number of leaves by the end of the vernalization treatment. Even though the developmental stages of the two groups were coordinated, the vernalization treatment can still affect the subsequent growth rates after vernalization. To eliminate this potential difference, spring lines with no vernalization requirement were included as controls in both vernalized and unvernalized treatments. The differences in heading time between the vernalized and unvernalized vernalization-insensitive spring lines were used to adjust the heading times of the other vernalized plants. The Δ*vrn-B1* mutants were used as controls in Figure 2A and the Δ*vrn-B1-*Δ*vrn2-*null mutant was used as a control in Figure 2C. Both control lines carry the functional vernalization-insensitive *VRN-A1* allele. The Δ*vrn-B1-*Δ*vrn2-*null mutant has the additional deletion of all functional copies of *VRN2* and differs from the Δ*vrn1-*Δ*vrn2-*null lines in the presence of the functional *VRN-A1* allele.

The experiment comparing the Δ*vrn1-* null and Δ*vrn1-vrn2-*null mutants (Figure 2C) was performed in the greenhouse (both mutants were grown under the same conditions); with an average day time and night time temperatures of 25°C and 17°C, respectively. Day length was extended to 16 h with high pressure sodium lights. Tissues for RNA extraction and qRT-PCR were collected at the same time (10:30 am) in all experiments to avoid potential confounding effects of circadian rhythms. Vernalization in this experiment was performed as described above.

To study gene expression in the apices (Figure 5B and 5D), we collected apices from *Δvrn1-*null mutants set 1 grown under long days in the greenhouse. Samples were collected before vernalization, six weeks after vernalization (4°C) and two weeks after the plants were moved from the cold to room temperature. At each sampling point, 90 apices were harvested and pooled into three replicates including 30 apices per replicate.

### Accession numbers

GenBank accession numbers are JX020745 to JX020760.

## Supporting Information

Figure S1 Positions and effects of the different mutations in the selected *VRN1* mutants. The G864A mutation in the Δ*vrn-B1_2619_* splice site mutant not only eliminated the splice site but also introduced a stop codon (TGA) at the same position. A) The black rectangles indicate *VRN1* exons. B) Chromatograms of wild type and homozygous mutant alleles. Note that mutations appear as single peaks, which indicates the presence of a single copy of each of the mutagenized genes.
(TIF)

Figure S2 qRT-PCR transcriptional profiles in the leaves of mutant set 2 (splice site Δ*vrn-A1*_2268_×Δ*vrn-B1*_2619_). A–B) *VRN1*, C–D) *FT,* E–F) *ZCCT2*. Left panels A, C and E) unvernalized plants. Right panels B, D, and F) vernalized plants. Blue shaded areas indicate vernalization at 4°C under long days. Δ*vrn-B1* mutants are indicated by blue lines (functional *Vrn-A1* allele, spring growth habit), Δ*vrn-A1* in red (functional *vrn-B1* allele, winter growth habit), and Δ*vrn1-*null in green. 0 wV: 3 weeks-old plants grown at 22°C/17°C (day/night) before vernalization, 3 wV: 3 weeks of vernalization, 6 wV: 6 weeks of vernalization, RT: two weeks after returning the vernalized plants to pre-vernalization conditions. A final sample was obtained from the flag leaves (FL) at heading time, which are indicated in days from sowing to heading (adjusted as indicated in Material and Methods in the vernalized plants). The X axis scale is not proportional to time and the Y scale is in fold-*ACTIN* values (number of molecules of target gene/number of molecules of *ACTIN*). Error bars are SE of the means from 8 biological replications.
(TIF)

Figure S3 qRT transcriptional profiles of *FUL2* and *FUL3* in the leaves. A–B) *FUL2,* C–D) *FUL3.* Left panels A and C) vernalized. Right panels B and D) unvernalized. The blue shaded area indicates vernalization at 4°C under long days. Δ*vrn-B1* mutants are indicated in blue (functional *Vrn-A1*, spring growth habit), Δ*vrn-A1* mutants in red (*vrn-B1*, winter growth habit), and Δ*vrn1-*null mutants in green. 0 wV: 3 weeks-old plants grown at 22°C day/17°C night before vernalization, 3 wV: 3 weeks of vernalization, 6 wV: 6 weeks of vernalization, RT: two weeks after returning the vernalized plants to pre-vernalization conditions. A final sample was obtained from the flag leaves (FL) at heading time, which are indicated in days from sowing to heading (adjusted in the vernalized plants as indicated in Material and Methods). The X axis scale is not proportional to time and the Y scale is in fold-*ACTIN* values. Error bars are SE of the means from 8 biological replications. The response of *FUL2* and *FUL3* to vernalization in the Δ*vrn1-*null mutants is shown in a more detail scale in Figure S4.
(TIF)

Figure S4 Transcriptional profiles of *FUL2, FUL3* and *FT* during and after vernalization in the leaves of Δ*vrn1-*null mutants set 2 (splice site mutants). A) *FUL3,* B) *FUL2* and *FT*. The blue shaded area indicates vernalization at 4°C under long days. 0 wV: 3 weeks-old plants grown at 22°C/17°C (day/night) immediately before vernalization, 3 wV: 3 weeks of vernalization, 6 wV: 6 weeks of vernalization, RT: two weeks after removing the plants from the cold and returning them to pre-vernalization conditions. The X axis scale is not proportional to time and the Y scale is in fold-*ACTIN* values. Error bars are SE of the means from 8 biological replications. Note the down-regulation of *FUL2,* and *FUL3* when plants were returned to room temperature, at the same time that the *ZCCT2* gene is up-regulated in the Δ*vrn1-*null mutants (Figure S2F).
(TIF)

Figure S5 Transcriptional profiles of *FUL2, FUL3* and *FT* during and after vernalization in the leaves of Δ*vrn1-*Δ*vrn2-*null mutant (no functional copies of *VRN1* or *VRN2*). A) *FUL3,* B) *FUL2* and *FT*. The blue shaded areas indicate vernalization at 4°C under long days. 0 wV: 3 weeks-old plants grown at 22°C/17°C (day/night) immediately before vernalization, 4 wV: 4 weeks of vernalization, 8 wV: 8 weeks of vernalization, RT: two weeks after removing the plants from the cold and returning them to pre-vernalization conditions. The X axis scale is not proportional to time and the Y scale is in fold-*ACTIN* values. Error bars are SE of the means from 8 biological replications. Compare the strong up-regulation of *FUL2,* and *FUL3* (>9-fold *ACTIN*) in the Δ*vrn1-*Δ*vrn2-*null plants after vernalization (RT) with the down-regulation observed at the same time point in the Δ*vrn1-*null mutants (functional *VRN2* gene) in Figure S4A and B.
(TIF)

Figure S6 Heading time and transcription profiles of isogenic lines of hexaploid wheat differing in *FT* expression levels. A) Heading time of plants grown under long days (16 h light/8 h dark). B–D) qRT-PCR transcription profiles in the leaves. The X axis scale is in weeks (w) and is not proportional to time. The Y scale is in fold-*ACTIN* values. B) *FT*, C) *FUL2* and D) *FUL3*. Abbreviations: JAG-OE = transgenic Jagger plants transformed with the Hope over-expressing *FT* allele [5], JAG = control winter wheat cultivar Jagger, CS-H7B = Hope 7B chromosome substitution carrying an over-expressing *FT* allele in CS, CS = control spring wheat cultivar Chinese Spring. Error bars are SE of the means from 8 biological replications. *** = *P*<0.0001.
(TIF)

Table S1 Primers and PCR conditions used for screening the TILLING population and for mutant detection.
(DOCX)

Table S2 Mutations resulting in truncations (splice sites and premature stop codon mutations) and amino acid changes with their respective Position-Specific Scoring Matrix (PSSM) and SIFT scores.
(DOCX)

Table S3 SYBR GREEN quantitative PCR primers for *FUL2* and *FUL3* and their respective amplification efficiencies.
(DOCX)
