# Supplementary figures and images for: Wheat TILLING Mutants Show That the Vernalization Gene VRN1 Down-Regulates the Flowering Repressor VRN2 in Leaves but Is Not Essential for Flowering
Source: PLoS Genet. 2012 Dec 13;8(12):e1003134. doi: 10.1371/journal.pgen.1003134 (PMC3521655; doi:10.1371/journal.pgen.1003134)

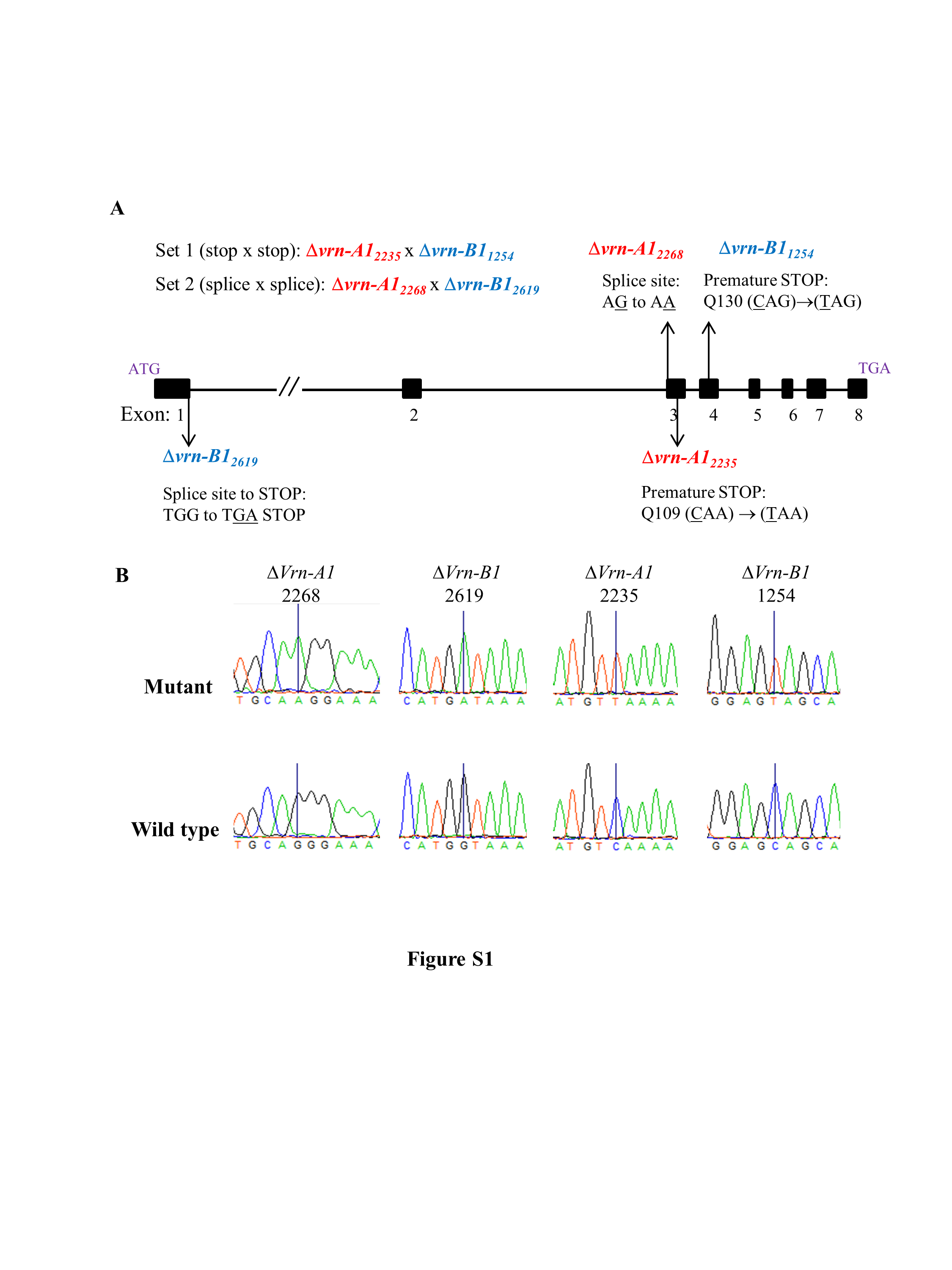

Supplement: Figure S1 — Positions and effects of the different mutations in the selected VRN1 mutants. The G864A mutation in the Δvrn-B12619 splice site mutant not only eliminated the splice site but also introduced a stop codon (TGA) at the same position. A) The black rectangles indicate VRN1 exons. B) Chromatograms of wild type and homozygous mutant alleles. Note that mutations appear as single peaks, which indicates the presence of a single copy of each of the mutagenized genes. (TIF) [file pgen.1003134.s001.tif]

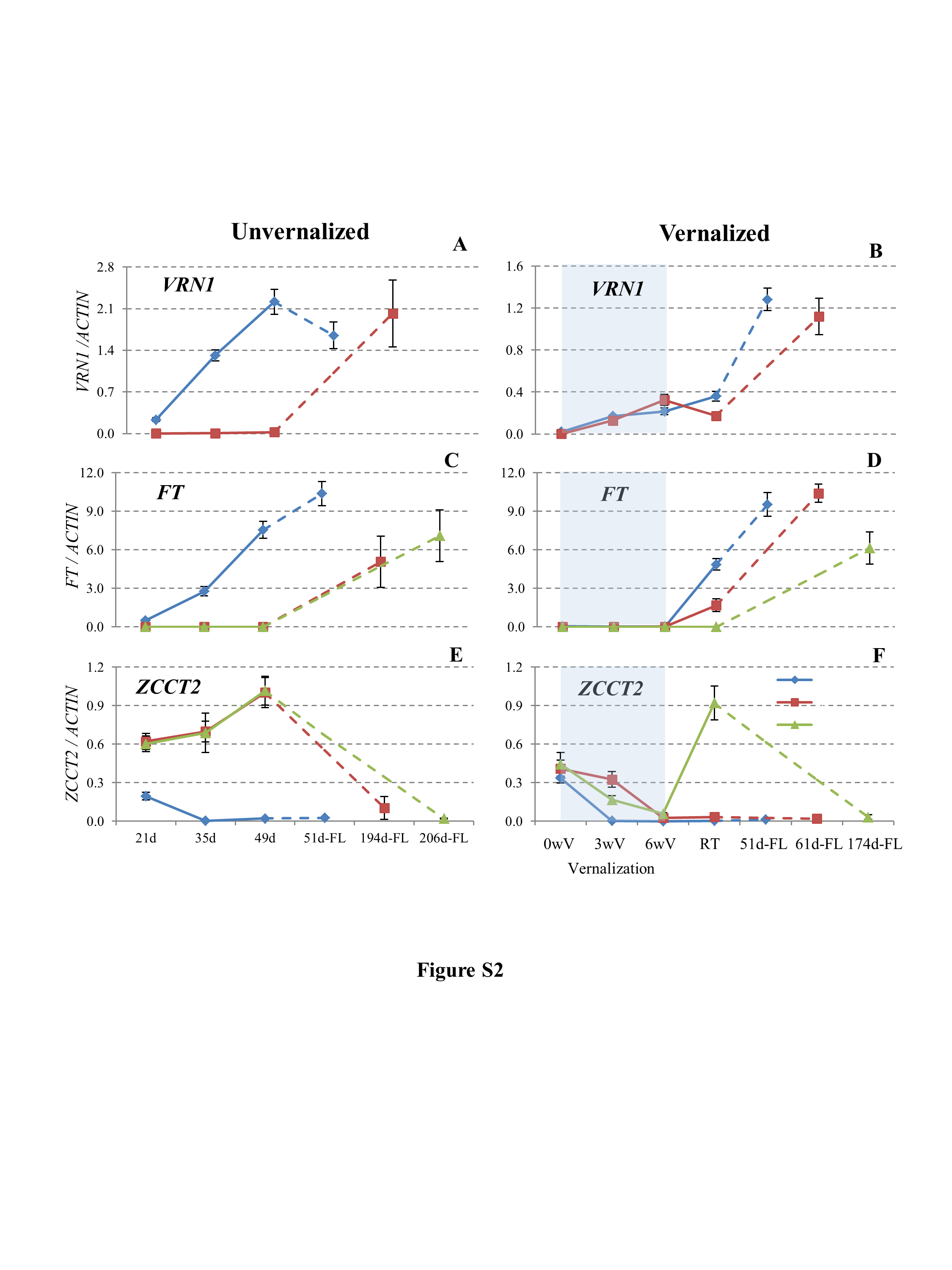

Supplement: Figure S2 — qRT-PCR transcriptional profiles in the leaves of mutant set 2 (splice site Δvrn-A1 2268×Δvrn-B1 2619). A–B) VRN1, C–D) FT, E–F) ZCCT2. Left panels A, C and E) unvernalized plants. Right panels B, D, and F) vernalized plants. Blue shaded areas indicate vernalization at 4°C under long days. Δvrn-B1 mutants are indicated by blue lines (functional Vrn-A1 allele, spring growth habit), Δvrn-A1 in red (functional vrn-B1 allele, winter growth habit), and Δvrn1-null in green. 0 wV: 3 weeks-old plants grown at 22°C/17°C (day/night) before vernalization, 3 wV: 3 weeks of vernalization, 6 wV: 6 weeks of vernalization, RT: two weeks after returning the vernalized plants to pre-vernalization conditions. A final sample was obtained from the flag leaves (FL) at heading time, which are indicated in days from sowing to heading (adjusted as indicated in Material and Methods in the vernalized plants). The X axis scale is not proportional to time and the Y scale is in fold-ACTIN values (number of molecules of target gene/number of molecules of ACTIN). Error bars are SE of the means from 8 biological replications. (TIF) [file pgen.1003134.s002.tif]

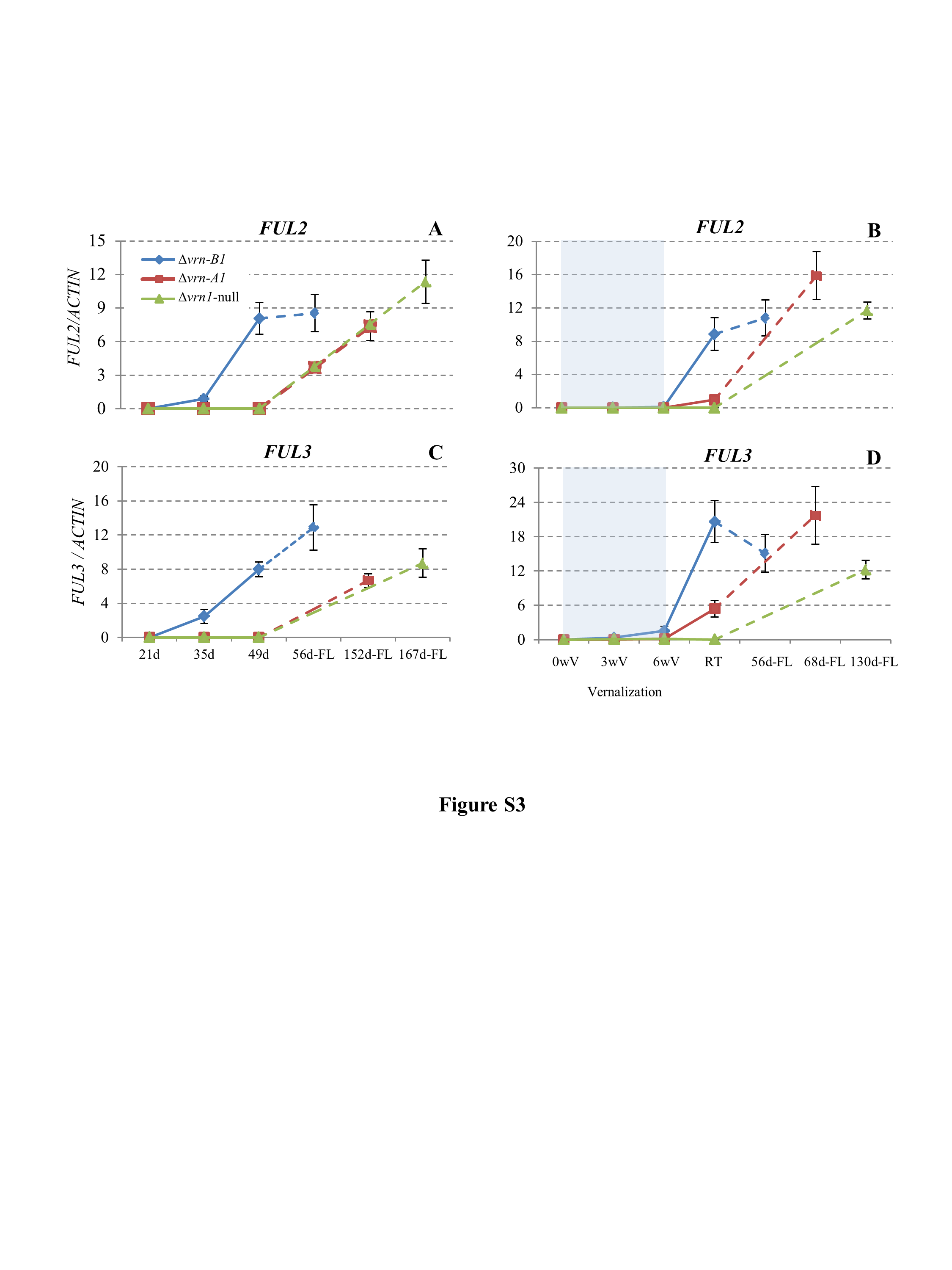

Supplement: Figure S3 — qRT transcriptional profiles of FUL2 and FUL3 in the leaves. A–B) FUL2, C–D) FUL3. Left panels A and C) vernalized. Right panels B and D) unvernalized. The blue shaded area indicates vernalization at 4°C under long days. Δvrn-B1 mutants are indicated in blue (functional Vrn-A1, spring growth habit), Δvrn-A1 mutants in red (vrn-B1, winter growth habit), and Δvrn1-null mutants in green. 0 wV: 3 weeks-old plants grown at 22°C day/17°C night before vernalization, 3 wV: 3 weeks of vernalization, 6 wV: 6 weeks of vernalization, RT: two weeks after returning the vernalized plants to pre-vernalization conditions. A final sample was obtained from the flag leaves (FL) at heading time, which are indicated in days from sowing to heading (adjusted in the vernalized plants as indicated in Material and Methods). The X axis scale is not proportional to time and the Y scale is in fold-ACTIN values. Error bars are SE of the means from 8 biological replications. The response of FUL2 and FUL3 to vernalization in the Δvrn1-null mutants is shown in a more detail scale in Figure S4. (TIF) [file pgen.1003134.s003.tif]

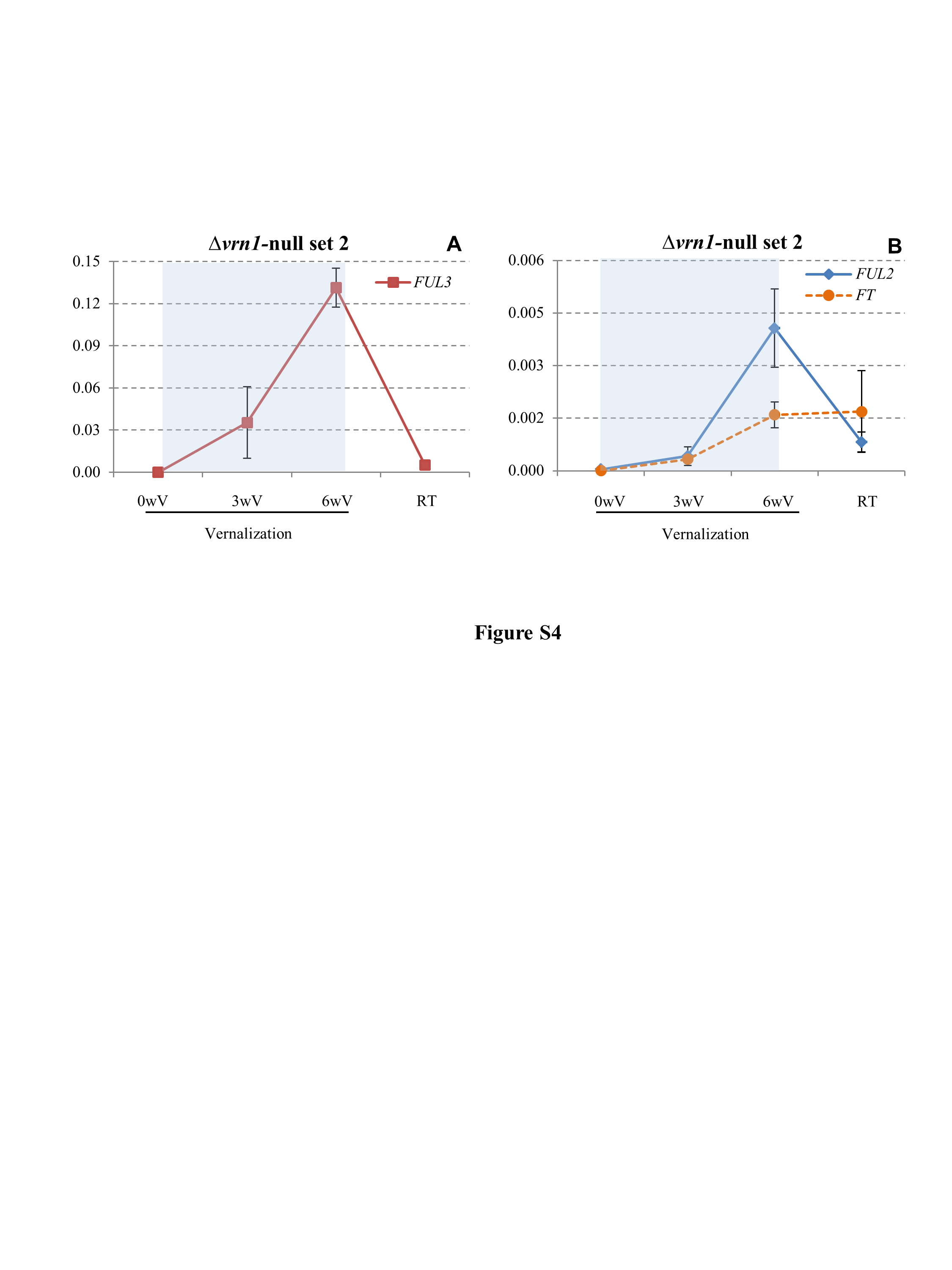

Supplement: Figure S4 — Transcriptional profiles of FUL2, FUL3 and FT during and after vernalization in the leaves of Δvrn1-null mutants set 2 (splice site mutants). A) FUL3, B) FUL2 and FT. The blue shaded area indicates vernalization at 4°C under long days. 0 wV: 3 weeks-old plants grown at 22°C/17°C (day/night) immediately before vernalization, 3 wV: 3 weeks of vernalization, 6 wV: 6 weeks of vernalization, RT: two weeks after removing the plants from the cold and returning them to pre-vernalization conditions. The X axis scale is not proportional to time and the Y scale is in fold-ACTIN values. Error bars are SE of the means from 8 biological replications. Note the down-regulation of FUL2, and FUL3 when plants were returned to room temperature, at the same time that the ZCCT2 gene is up-regulated in the Δvrn1-null mutants (Figure S2F). (TIF) [file pgen.1003134.s004.tif]

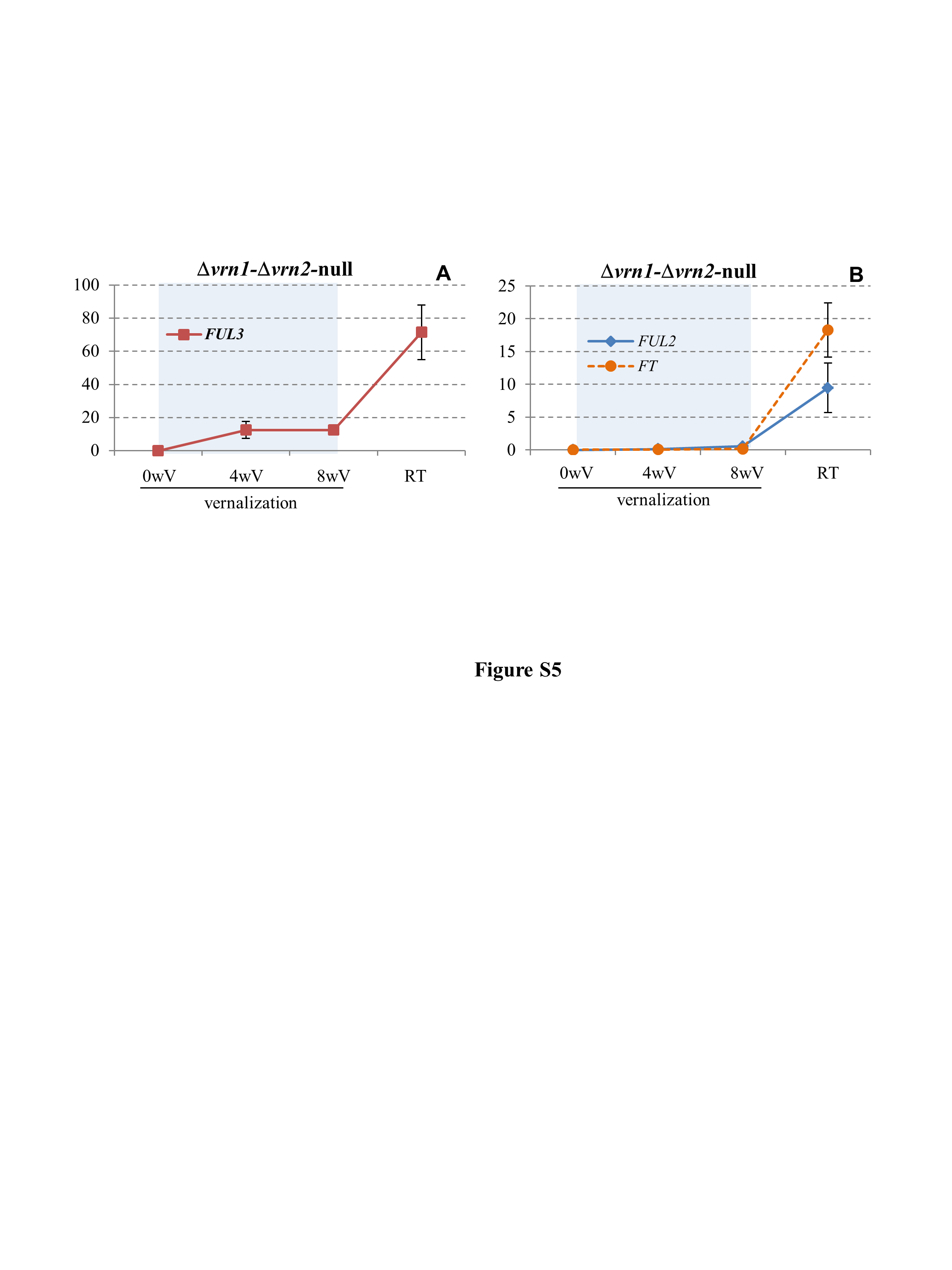

Supplement: Figure S5 — Transcriptional profiles of FUL2, FUL3 and FT during and after vernalization in the leaves of Δvrn1-Δvrn2-null mutant (no functional copies of VRN1 or VRN2). A) FUL3, B) FUL2 and FT. The blue shaded areas indicate vernalization at 4°C under long days. 0 wV: 3 weeks-old plants grown at 22°C/17°C (day/night) immediately before vernalization, 4 wV: 4 weeks of vernalization, 8 wV: 8 weeks of vernalization, RT: two weeks after removing the plants from the cold and returning them to pre-vernalization conditions. The X axis scale is not proportional to time and the Y scale is in fold-ACTIN values. Error bars are SE of the means from 8 biological replications. Compare the strong up-regulation of FUL2, and FUL3 (>9-fold ACTIN) in the Δvrn1-Δvrn2-null plants after vernalization (RT) with the down-regulation observed at the same time point in the Δvrn1-null mutants (functional VRN2 gene) in Figure S4A and B. (TIF) [file pgen.1003134.s005.tif]

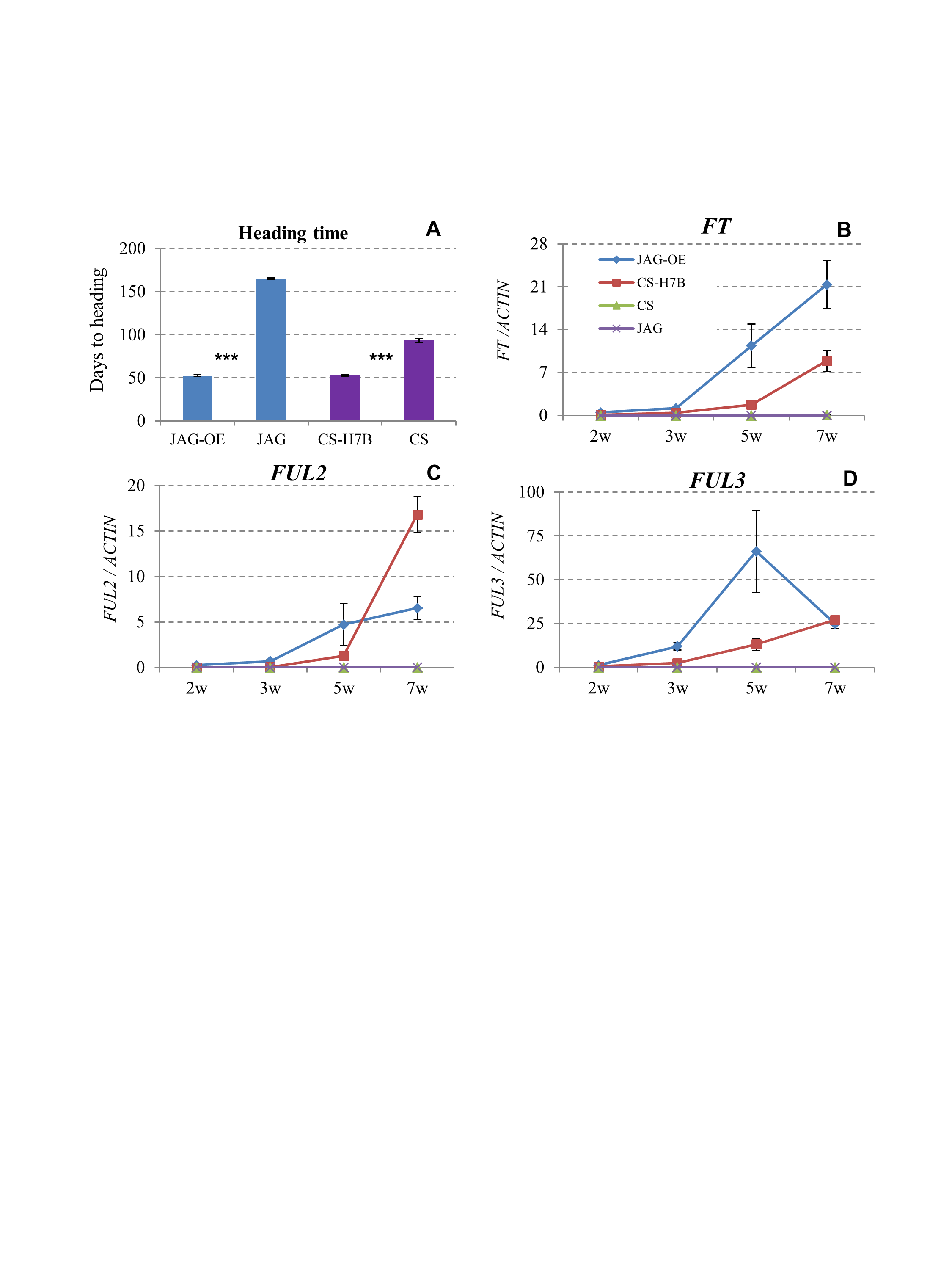

Supplement: Figure S6 — Heading time and transcription profiles of isogenic lines of hexaploid wheat differing in FT expression levels. A) Heading time of plants grown under long days (16 h light/8 h dark). B–D) qRT-PCR transcription profiles in the leaves. The X axis scale is in weeks (w) and is not proportional to time. The Y scale is in fold-ACTIN values. B) FT, C) FUL2 and D) FUL3. Abbreviations: JAG-OE = transgenic Jagger plants transformed with the Hope over-expressing FT allele [5], JAG = control winter wheat cultivar Jagger, CS-H7B = Hope 7B chromosome substitution carrying an over-expressing FT allele in CS, CS = control spring wheat cultivar Chinese Spring. Error bars are SE of the means from 8 biological replications. *** = P<0.0001. (TIF) [file pgen.1003134.s006.tif]
