# Supplementary material for: Wheat TILLING Mutants Show That the Vernalization Gene VRN1 Down-Regulates the Flowering Repressor VRN2 in Leaves but Is Not Essential for Flowering
Source: PLoS Genet. 2012 Dec 13;8(12):e1003134. doi: 10.1371/journal.pgen.1003134 (PMC3521655; doi:10.1371/journal.pgen.1003134)
Supplement: Table S1 — Primers and PCR conditions used for screening the TILLING population and for mutant detection. (DOCX) [file pgen.1003134.s007.docx]

**Table S1**. Primers and PCR conditions used for screening the TILLING population and for mutant detection.

| **Gene name** | **Target** | **Primer name** | **Primer sequence (5' to 3')** | **Product  size (bp)** | **Annealing  Temp (⁰C)** | **Extension  time** | **Enzyme** |
| --- | --- | --- | --- | --- | --- | --- | --- |
| ***Screening primers*** | |  |  |  |  |  |  |
| *VRN-A1* | Exon 1 | V1A-5PRIME-F1 | GAAAGGAAAAATTCTGCTCG | 713 | 59* | 1 m 30 s | - |
|  |  | V1-INT1R | GCAGGAAATCGAAATCGAAG |  |  |  |  |
| *VRN-A1* | Exon 3 to 6 | VA1-3PRIME-F2 | GCCTATTTGTAGCATTTCTGTCATT | 847 | 60* | 1 m 30 s | - |
|  |  | VA1-3PRIME-R3 | GGAAGGGACACGGACCTC |  |  |  |  |
| *VRN-B1* | Exon 1 | V1B-5PRIME-F2 | CTAGACGGCCCAAAACAAGA | 1076 | 61* | 1 m 30 s | - |
|  |  | V1B-5PRIME-R1 | ACGGATGGAAACAGCTACCGA |  |  |  |  |
| *VRN-B1* | Exon 3 to 6 | VB1-3PRIME-F2 | GCCTATTTGTAGCATTTCCGTCATG | 853 | 58* | 1 m 30 s | - |
|  |  | VB1-3PRIME-R3 | GGAAGGGACAGGGATCTG |  |  |  |  |
| ***Mutant detection primers*** | |  |  |  |  |  |  |
| *VRN-A1* | T4-2235 & | VA1-3PRIME-F2 | Same as above | 446 | 56 | 50 s | *Bsg*I |
|  | T4-2268 | VRN-A1-CAPS-R2 | GAACATCTCAGTCTAGAATCTGAT |  |  |  |  |
| *VRN-B1* | T4-1051 | VRN-B1-CAPS-F1 | CGCCTCACCCAACCACCTGAC | 482 | 62 | 50 s | *Mbo*II |
|  | T4-2619 | V1B-5PRIME-R1 | Same as above |  |  |  | *Bsl*I |
| *VRN-B1* | T4-1254 | VB1-3PRIME-F2 | Same as above | 451 | 62 | 50 s | *Mwo*I |
|  |  | VRN-B1-CAPS-R1 | GTGAATATCTCAGTCTAGAATCGGAC |  |  |  |  |

*94⁰C for 5 m, 94⁰C for 30 s, 12 cycles of initial touch down (-0.5⁰C per cycle), followed by 40 cycles of normal PCR, final extension time of 7 min at 72⁰C.

The underlined base is a T to G mismatch introduced in the VRN-B1-CAPS-R1 primer to increase B-genome specificity.
