# Supplementary material for: Wheat TILLING Mutants Show That the Vernalization Gene VRN1 Down-Regulates the Flowering Repressor VRN2 in Leaves but Is Not Essential for Flowering
Source: PLoS Genet. 2012 Dec 13;8(12):e1003134. doi: 10.1371/journal.pgen.1003134 (PMC3521655; doi:10.1371/journal.pgen.1003134)
Supplement: Table S2 — Mutations resulting in truncations (splice sites and premature stop codon mutations) and amino acid changes with their respective Position-Specific Scoring Matrix (PSSM) and SIFT scores. (DOCX) [file pgen.1003134.s008.docx]

**Table S2**. Mutations resulting in amino acid changes and truncations (splice sites and premature stop codon mutations).

| **Gene (region)** | **Line** | **GenBank** | **Nucleotide**  **change ^1^** | **Amino acid**  **change ^1^** | **PSSM ^2^** | **SIFT ^2^** |
| --- | --- | --- | --- | --- | --- | --- |
| *VRN-A1* (exon 1) | T4-2430 | JX020745 | G436A | V6M | 2.5 | 0.02 |
| *VRN-A1* (exon 3-6) | T4-2268 | JX020746 | G67A | Splice junction | - | - |
|  | T4-2235 | JX020747 | C128T | Q109* | - | - |
|  | T4-127 | JX020748 | C226T | L112F | 7.3 | 0.12 |
|  | T4-118 | JX020749 | G238A | D116N | 7.9 | 0.17 |
|  | T4-612 | JX020750 | G293A | S134N | -2.6 | 0.77 |
|  | T4-524 | JX020751 | G514A | Splice junction | - | - |
|  | T4-381 | JX020752 | G542A | E153K | -13.8 | 1 |
|  | T4-2365 | JX020753 | G708A | E158K | -5.1 | 0.29 |
| *VRN-B1* (exon 1) | T4-1051 | JX020754 | G694A | V6M | 2.5 | 0.02 |
|  | T4-2619 | JX020755 | G864A | Splice junc. & stop | - | - |
| *VRN-B1* (exon 3-6) | T4-2236 | JX020756 | G104A | V102I | -3.3 | 0.42 |
|  | T4-2292 | JX020757 | G237A | D116N | 7.9 | 0.17 |
|  | T4-1254 | JX020758 | C279T | Q130* | - | - |
|  | T4-1134 | JX020759 | C535T | S150F | 10.9 | 0 |
|  | T4-260 | JX020760 | C546T | L154F | 19.1 | 0.02 |

**^1^**The first and last letters in the nucleotide and amino acid change columns present the wild type and the mutant alleles respectively. The number in between the two letters indicates the position of the mutation relative to the 5’ start position of the sequence identified by the GenBank accession. For the amino acid changes the middle number indicates the position of the amino acid substitution relative to the first methionine of the predicted protein.

**^2^** Mutations with high Position-Specific Scoring Matrix (PSSM >10) and/or low SIFT scores (<0.05) encode amino acid changes that are predicted to have significant effects on the protein function. PSSM and SIFT scores are not reported for premature stop codon and splice junction mutations.
