# Supplementary material for: Wheat TILLING Mutants Show That the Vernalization Gene VRN1 Down-Regulates the Flowering Repressor VRN2 in Leaves but Is Not Essential for Flowering
Source: PLoS Genet. 2012 Dec 13;8(12):e1003134. doi: 10.1371/journal.pgen.1003134 (PMC3521655; doi:10.1371/journal.pgen.1003134)
Supplement: Table S3 — SYBR GREEN quantitative PCR primers for FUL2 and FUL3 and their respective amplification efficiencies. (DOCX) [file pgen.1003134.s009.docx]

**Table S3**. SYBR GREEN® quantitative PCR systems for *FUL2* and *FUL3.*

| **Name** | **Forward (5’ → 3’)** | **Reverse (5’ → 3’)** | **Amplification efficiency** |
| --- | --- | --- | --- |
| *FUL2* | CCATACAAAAATGTCACAAGC | TTCTGCCTCTCCACCAGTTC | 98.4% |
| *FUL3* | ATGGATGTGATTCTTGAACG | AGTTGCCTTTGACTCTTCTG | 96.1% |
